# A Functional Bayesian Model for Hydrogen–Deuterium Exchange Mass Spectrometry

**DOI:** 10.1021/acs.jproteome.3c00297

**Published:** 2023-08-15

**Authors:** Oliver M. Crook, Nathan Gittens, Chun-wa Chung, Charlotte M. Deane

**Affiliations:** † Department of Statistics, University of Oxford, Oxford OX1 3LB, United Kingdom; ‡ Structural and Biophysical Sciences, GlaxoSmithKline R&D, Stevenage SG1 2NY, United Kingdom

**Keywords:** Bayesian, mass spectrometry, hydrogen−deuterium exchange, temporal, MCMC

## Abstract

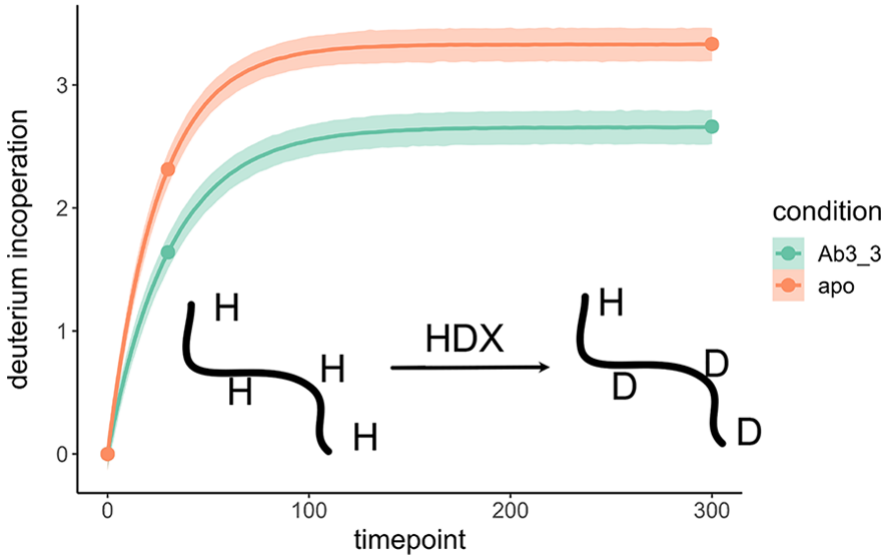

Proteins often undergo structural perturbations upon binding to other proteins or ligands or when they are subjected to environmental changes. Hydrogen–deuterium exchange mass spectrometry (HDX-MS) can be used to explore conformational changes in proteins by examining differences in the rate of deuterium incorporation in different contexts. To determine deuterium incorporation rates, HDX-MS measurements are typically made over a time course. Recently introduced methods show that incorporating the temporal dimension into the statistical analysis improves power and interpretation. However, these approaches have technical assumptions that hinder their flexibility. Here, we propose a more flexible methodology by reframing these methods in a Bayesian framework. Our proposed framework has improved algorithmic stability, allows us to perform uncertainty quantification, and can calculate statistical quantities that are inaccessible to other approaches. We demonstrate the general applicability of the method by showing it can perform rigorous model selection on a spike-in HDX-MS experiment, improved interpretation in an epitope mapping experiment, and increased sensitivity in a small molecule case-study. Bayesian analysis of an HDX experiment with an antibody dimer bound to an E3 ubiquitin ligase identifies at least two interaction interfaces where previous methods obtained confounding results due to the complexities of conformational changes on binding. Our findings are consistent with the cocrystal structure of these proteins, demonstrating a bayesian approach can identify important binding epitopes from HDX data. We also generate HDX-MS data of the bromodomain-containing protein BRD4 in complex with GSK1210151A to demonstrate the increased sensitivity of adopting a Bayesian approach.

## 1 Introduction

Protein structures can be perturbed due to alterations in their context, and HDX-MS is a powerful technique to examine these perturbations.^1−6^ When a protein is incubated in heavy water its amide hydrogens exchange with deuterium at a context-specific rate.^7^ This rate of exchange will be in accordance with Linderstrom-Lang theory^8^ and is also affected by solvent accessibility, topological flexibility, amino-acid content, secondary structure and conformal heterogeneity.^9−11^ Since deuterium is heavier than hydrogen, we can measure rates of incorporation by examining mass-shifts and isotopic expansion using bottom-up mass spectrometry.^5^ To mediate complex protein kinetics HDX-MS is performed over a time-course, examining the deuterium incorporated at different exposure times of the sample to heavy water.^12^

We recently showed that using tools from functional data analysis and empirical Bayes analysis could increase power, reduce false positives, and improve interpretation of HDX-MS experiments when compared to *t* test and linear mixed models.^12^ Furthermore, that functional data analysis approach could be applied to large epitope mapping experiments, where there were no previously rigorous statistical tools. This approach fits either logistic or Weibull kinetics to HDX-MS data; then, to assess the quality of fits, an F-statistic is computed. This statistic is then moderated using an empirical Bayes approach.^12^

However, to apply our earlier approach^12^ required restrictive assumptions on the kinetics, which reduces power. Here, we recast our analysis in the Bayesian framework, which allows us to further increase the flexibility of the model.^13^ Bayesian analysis has a number of further advantages.^13^ First, it allows us to shrink residuals toward zero, meaning that differences between HDX kinetics are easier to identify. Second, it allows us to regularize the inferred parameters, allowing us to fit functional models with more parameters than observations. Third, model fitting is less sensitive to initialization and so produces more reliable results.^13^ In addition, Bayesian analysis allows us to quantify uncertainty using probability distributions, allowing us to report the confidence in our results.^14^ This also allows us to compute quantities that are impossible to obtain without using a Bayesian approach, for example, the probability that a deuterium difference exceeds a particular value. A Bayesian method for HDX-MS data has previously been developed Saltzberg et al.,^15^ but the focus of that work is on determining residue-level information rather than statistical differences at the peptide level. Furthermore, that approach employs clustering before significance testing, which means the hypothesis tested is conditioned on the data, which violates selective inference protocols and hence inflates false positives.^16^

First, we show that our approach is consistent: controlling false positives and obtaining true positives in simulations. We then examine competing models for HDX and show that when there are EX1 dynamics, a Weibull model is preferred over a logistic model for HDX data. We characterize this in a spike-in HDX experiment showing a strong preference for almost all peptides to follow Weibull-type kinetics.^17^ However, we find no evidence to support a functional mixed-model for HDX. Finally, we perform epitope mapping in HOIP-RBR^18^ showing that we can increase the number of findings in these experiments when compared to an empirical Bayes approach.^12^ In particular, we were previously unable to identify the binding epitope of dAb3 from the HDX data using any previous statistical method; however, our Bayesian analysis of this HDX experiment suggests three possible binding interfaces that are supported by the cocrystal structure of the dAb3 dimer and HOIP-RBR. In this case, we present an array of new visualizations and computational techniques afforded by taking a probabilistic approach. Finally, we compare our previous approach with our Bayesian analysis on an application to BRD4 in complex with selective small molecule I-BET151 and demonstrate that superior sensitivity can be maintained even as data points are ablated.

## 2 Materials and Methods

### 2.1 Preliminaries

In hydrogen–deuterium exchange mass spectrometry, we observe isotopic distributions for *i* = 1, ..., and *n* peptides at different exposure times *t*_1_, *t*_*m*_ to heavy water (D_2_O). The isotope distributions are a set of  pairs reporting the relative intensities of each peptide isotope. These isotope distributions are frequently summarized by the intensity weighted mean of the , which we write as  and is referred to as the centroid. Since deuterium is heavier than hydrogen, deuterium interoperation leads to positive shifts in  and we monitor this change over time and with respect to the state. In most scenarios data are replicated, so we observe replicates *r* = 1, ..., *R* and, potentially, a number of conditions/covariates denoted *c* = 1, .., *C*. The observations are then
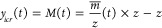
1where *z* denotes the charge of the precursor ion. It is typical to normalize such that mass *M*(0) = 0.

### 2.2 Bayes’ Theorem and Hypothesis Testing

Here, we summarize Bayesian inference and hypothesis testing.^13,19^ We first note that multiplicity is implicitly controlled via prior distributions on the parameters as well as explicitly via the prior model probabilities. Prior information on the parameters imparts a number of advantages, including shrinkage of residuals toward 0, regularization of parameters, and more stable algorithmic inference. Furthermore, a Bayesian analysis allows us to sample from the posterior distribution of quantities of interest, and using this probability distribution is central to quantifying uncertainty.

Bayesian inference requires several quantities to be specified. The first is a statistical model , with parameters θ, of the observed data *y*. After specifying a prior distribution for the parameters, denoted , and given observed data *y*, Bayes’ theorem tells us we can update the prior distribution to obtain the posterior distribution using the following formula:
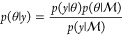
2The quantity  is referred to as the marginal likelihood, and is obtained by marginalizing θ:
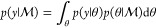
3The task of hypothesis testing can be reformulated as a model selection problem. We write  to denote the model associated with the null hypothesis; while the alternative hypothesis is associated with model . Thus, hypothesis testing can be phrased as selecting between two competing models.

To perform model selection, we are interested in the posterior model probability, given the data:^20^
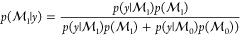
4The relative plausibility of two model is quantified through the posterior odds, which is the prior odds multiplied by the Bayes factor:^21^
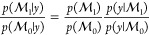
5These quantities are challenging to compute, because of the marginal likelihoods required. Note that the prior on the parameters penalizes additional model complexity. Given that the marginal likelihood is only analytically available for relatively simple models, we approximate it using Bridge sampling (see the following section).^22,23^ Though a number of other methods are available such as path sampling,^24^ importance sampling,^25^ harmonic mean sampling,^24^ nested sampling,^26,27^ and Laplace’s approximation.^28^

Finally, we assume that the null model is more probable than the alternative, controlling multiplicity.^29^ Thus, we set  and .

### 2.3 Bridge Sampling for Marginal Likelihoods

We provide brief details on bridge sampling to compute the marginal likelihood.^22,23^ We first observe the following identity:
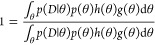
6where *g*(θ) is the proposal distribution and the bridge function is denoted by *h*(θ). Then through multiplication of both sides by *p*(*D*), we obtain
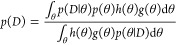
7The numerator can be written as an expectation with respect to *g*(θ), while the denominator is an expectation with respect to the posterior *p*(θ|*D*). Hence, we observe that
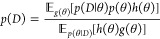
8This allows to approximate the marginal likelihood using the following estimator:
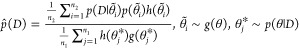
9The important observations are that we need samples from the proposal distribution *g*(θ) and the posterior *p*(*D*|θ). The samples from the posterior are simply obtained from the MCMC algorithm used in model inference. For the proposal distribution to have good empirical performance it should have the same support as the posterior, and so a normal proposal with moments matched to the posterior distribution is suitable. We have yet to mention how to decide on the bridge function *h*(θ). The so-called optimal bridge function takes the following form:^23^
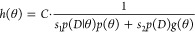
10where *s*_1_ = *n*_1_/(*n*_2_ + *n*_1_) and *s*_2_ = *n*_2_/(*n*_1_ + *n*_2_). The constant *C* is irrelevant because it cancels in the estimator ratio. The bridge function is optimal in the sense that in minimizes relative mean-squared error. However, a clear issue with this choice of bridge function is that it depends on the quantity that we are trying to estimate *p*(*D*). The resolution is to make an initial guess for the marginal likelihood *p*^(0)^(*D*) and iteratively update accordingly. The update equation is given by
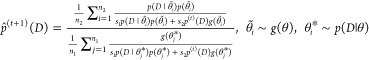
11The above iterative estimator demonstrates why bridge sampling is robust to the tail behavior of the proposal distribution relative to the posterior distribution—a property not held by other methods such as importance sampling.

### 2.4 Prior and Posterior Predictive Checks

In this section, we summarize prior and posterior predictive checks, which allow us to build high quality generative and predictive models.^14,30,31^ Given a likelihood and prior, we can simulate data *ỹ*.^32^ First, the parameters of the likelihood were sampled from the prior, and then given these parameters, data were sampled from the model:
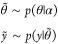
12This leads us to define the *prior predictive distribution*:
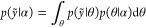
13See Gelman, Simpson, and Betancourt^32^ for advantages of working with the prior predictive distributions. To define the posterior predictive distribution, we can then sample new data by first sampling parameters from the posterior distribution and then again sampling from the likelihood:
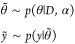
14This leads to the posterior predictive distribution:
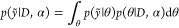
15Once we have data generated from a predictive distribution, we can generate a summary of this data *S*(*ỹ*). We can then visually compare the summary of an ensemble of simulated data sets *S*(*ỹ*_1_), *S*(*ỹ*_2_), ..., *S*(*ỹ*_*n*_), with the summary of the observed data *S*(*y*) to identify model deficiencies.

### 2.5 Out-of-Sample Predictive Performance

Another approach to evaluate the quality of a Bayesian model is to examine the out-of-sample predictive accuracy from the fitted model. We can employ (approximate) leave-one-out cross validation (LOO-CV) with log predictive density as the utility function:^33^
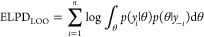
16The above equation is the leave-one-out predictive density given the observed data with the *i*^th^ observation removed, summed over the observations. This quantity is estimated using Pareto smoothed importance sampling (PSIS).^34^

### 2.6 Modeling

Here, we state the proposed models considered in this paper. This includes a logistic model, a Weibull model, and a random-effects model. In each of these cases, we consider a model  in which a single function is posited and blinded to any contexts, covariates or treatment effects. Meanwhile, we also consider allowing the parameters of the model, , to be context-specific.

For the logistic model, we assume
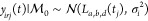
17and
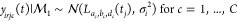
18where *c* denotes the context. The mean logistic model is given by
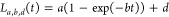
19To complete the specification of the model, we need to specify priors. The first parameter is *d*, which can be interpreted as an intercept, which should be symmetric and concentrated around 0. The parameter *a* represents the plateau of the model (dominant for large values of *t*). This parameter is also required to be positive. The parameter *b* is a shape parameter and is also required to be positive. The standard deviation σ also must be positive; furthermore, we wish to penalize this parameter to avoid concentrating on models that are completely explained by noise. These considerations lead to the following hierarchical structure:
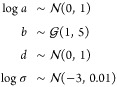
20These priors were chosen using iterative prior predictive checks, and the quality of posterior inference was examined using posterior predictive checks (see the Supporting Information).

We also consider a Weibull model for HDX-MS data, which allows more flexibility in the temporal kinetics. As for the logistic model, we posit
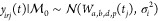
21and
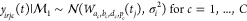
22where *c* denotes the context. The mean logistic model is given by
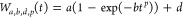
23The prior construction is the same for the logistic model, but in addition, we propose the following:
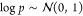
24Finally, we consider a functional random-effects Weibull model by allowing random plateaus using a replicate level grouping nested within condition. That is, the parameter *a* is modeled as follows, denoting *r* for replicate
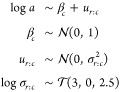
25

### 2.7 Inference and Implementation

Model and parameter inference is performed using Markov-chain Monte Carlo (MCMC); in particular, we use the No U-Turn Sampler (NUTS), a variant of Hamiltonian Monte Carlo, as an implement in the probabilistic programming language stan.^35,36^

### 2.8 Previous Approaches

We compare to an empirical Bayes functional modeling approach.^12^ Briefly, Weibull or logistic functional models are fitted to the time-dependent HDX kinetics. By fitting a null model that is blinded to the covariates and an alternative hypothesis that uses independent models for each context, we are able to compute the residual sum of squares for each model. From this, we can compute an F-statistic with the appropriate degrees of freedom. To borrow power, an empirical Bayes method is applied, allowing the variances to be shrunk toward a pooled variance. This stabilizes variance estimates and improves power by computing a *moderated* F-statistic. The *p*-values are then computed from the appropriate F-distribution and corrected for multiple testing using the Benjamini–Hochberg procedure.

### 2.9 Performance Metrics

To compare the Bayesian approach with the empirical Bayes functional method, we use the area-under-curve of receiver operating characteristics (AUCROC). That is, the area under a curve by plotting the true positive rate (TPR) against the false positive rate (FPR). An AUCROC of 1 indicates perfect performance, while 0.5 denotes random performance. To assess the calibration of probabilities from the Bayesian approach, we computed the Brier score. A Brier score of 0 indicates probabilities that are perfectly calibrated and a Brier score of 1 indicates probabilities that display no calibration.^37^

### 2.10 Simulation Study

This section describes our proposed simulation study, as in ref (12). We begin by sampling, uniformly at random, the length of the peptide between 5 and 25. The sampled number is the number of amino acids in the peptide, and we sample that number of amino acids from the 20 canonical amino acids with replacement. We then define time points at which to obtain data: *T* = {*t*_1_, ..., *t*_*m*_}, with *t*_1_ = 0 and *t*_*i*_ < *t*_*j*_ for *i* < *j*. For time *t*_1_, we simulate the undeuterated isotope distribution using a binomial modal. For a subsequent time point *t*_*i*_ we sample the percentage incorporation by first sampling from a *m* – 1-variate Dirichlet distribution with concentration parameter α, where α_*i*_ = 20/(*i* – 1). From this, we obtain a vector π, which sums to 1. We used the cumulative distribution of π as the schedule of incorporations. That is, the incorporation at *t*_*i*_*D*_*i*_ = ∑ _*r* = 1_^*i*–1^π_*r*_ for *i* > 1. This ensures that incorporation is nondecreasing in time. Only exchangeable amides are considered for deuterium interoperation with Prolines and the two N-terminal residues disregarded. To simulate the effect of a condition, for each time point, we sample an indicator *z*_*t*_*i*__ ∈ {0, 1} such that the *p*(*z*_*t*_*i*__ = 0) = 0.95. If *z*_*t*_*i*__ = 1, then we resample the incorporation amount and continue on the simulation process. This ensures that roughly 95% of the scenarios have no effect with respect to the condition. A binomial model is used to generate deuterated spectra, where the exchangeable hydrogens are randomly replaced with deuterium according to the incorporation percentage. The isotope distribution simulations are repeated *R* times to allow for replicates. Centroids summarizing the average peptide mass are then computed from the isotope distribution. In all cases, we simulate 100 measured peptides. We perform simulation scenarios as follows

- (Scenario A) 4 time points, 3 replicates, and 2 conditions
- (Scenario B) 4 time points, 2 replicates, and 2 conditions
- (Scenario C) 5 time points, 2 replicates, and 2 conditions

### 2.11 HDX-MS of BRD4 with i-BET151

A 6xHis-BRD4(1-477) construct was expressed and purified as previously described.^38^ For HDX labeling experiments, a 2.5 μM stock of BRD4 protein (12.5 pmol) was prepared by dilution in a reference buffer of 50 mM MOPS, 150 mM NaCl, pH 7.2. The inhibitor-bound and apo forms were prepared by the addition of i-BET151 (for a final concentration of 25 μM) or an equivalent volume of DMSO (2% final concentration) and preincubated for at least 30 min at 1 °C.

The reaction was initiated by a 12-fold dilution of 5 μL protein sample (12.5 pmol) in a labeling buffer (50 mM MOPS, 150 mM NaCl, pD 7.2 (pHread = 6.8 with standard calomel electrode)) at 20 °C using an automated sample handling workflow (LEAP HDX PAL, Trajan Scientific). Labeling times were sampled at 0, 15, 60, 600, 3600, and 14 400 s in triplicate. Protein samples were quenched and denatured by an equal volume of quench solution (6 M guanidine hydrochloride, 400 mM sodium phosphate pH 2.2, 2% formic acid) for 1 min at 1 °C and immediately injected onto an immobilized nepenthesin-2 column (2.1 mm × 20 mm, Affipro, CZ). The resultant peptides collected on a precolumn trap (UPLC BEH C18 Vanguard, Waters) for 4 min with 0.2% formic acid and 0.03% TFA in H_2_O at a flow rate of 100 μL/min. Peptides were then eluted by liquid chromatography (1.7 μm UPLC BEH C18 column, 1.0 × 50 mm dimensions, 130 Åpore size, Waters) for 12 min at a flow rate of 20 μL/min at 0 °C, over a gradient of 11–40% of 0.2% formic acid in MeCN before ramping to 98% for a further 3 min, and a 4 min sawtooth gradient cleaning cycle. A LeuEnk and GluFib lock solution was coinjected as an internal standard. Data were acquired on a Synapt G2-Si high definition mass spectrometer (Waters) in the data-independent HDMS acquisition mode. For fully labeled control experiments, BRD4 was labeled for 1 h in 6 M *d*_5_-guanidine deuteriochloride diluted in a labeling buffer, before quenching as described above. Samples for the 100% D control experiments were handled manually.

Peptide mapping experiments were completed in a separate experiment with a 25 pmol injection of protein diluted in the unlabeled reference buffer, and subsequently treated as above, using a HDMSe acquisition mode. Peptide lists from the peptide mapping experiments were generated in Protein Lynx Global Server 3.0 software. The peptide lists generated were subsequently imported into HDExaminer v2.5.0 (Sierra Analytics, Modesto, CA) and subject to further filtering (peptide length <25; PLGS Score >6.5; products per amino acid > 0.3; Δppm < ±10) and analyzed to determine deuterium uptake. Only peptides containing data with adequate intensity and reliable isotope distributions for all time points and both states were preserved.

### 2.12 Differential Solvent Accessibility Analysis

To perform differential solvent accessibility analysis, we computed the accessible solvent area (ASA) for each residue of the unbound and bound forms of HOIP-RBR. We then took the square root of these values so that the results were on the linear scale and computed the difference. Finally, results were converted to *z*-scores. Significant differences in ASA were identified by computing the local false discovery rate (fdr).^39^ We recall that the fdr is the probability that there is no change in the observed differences, for a given test statistic; that is, fdr(*z*_*i*_) = *P*(*z*_*i*_ = 0| *Z* ≤ *z*_*i*_). A threshold of 0.01 was set to declare a difference.

## 3 Results

### 3.1 Model Summary

We begin with a narrative description of our model, with technical details given in the Materials and Methods. We propose modeling HDX-MS data using functional models (time-dependent curves). We choose to use smooth parametric models for the modeling, using either a logistic or Weibull-type model, depending on the expected kinetics. We then place prior distributions on the parameters of these models, and Bayes’ theorem tells us that after observing data we can update these prior distributions into posterior distributions.^13^ These posterior distributions quantify the uncertainty in the model parameters. The Bayesian framework allows us to compute probability distributions as a function of time as well as the probability of a model given the data. For example, we can compute whether covariate dependent models are preferred over models that are blind to the covariates. Covariates in this scenario would be any conditions, states, or general quantities that could potentially perturb HDX kinetics. This preference is quantified through the posterior probability of a particular model. In this paper, we introduce several new quantities, such as the probability of deuterium incorporation being above a certain value. These quantities are difficult to compute in other statistical frameworks. In addition, the prior distribution acts as regularization for our model parameters and hence rules-out improbable parameters; this stabilizes the inference in our model. For a review of Bayesian methods applied to proteomics data, we refer to Crook, Chung, and Deane.^40^

### 3.2 Simulations Show the Bayesian Approach Is Powerful and Calibrated

First, we characterize the performance of our approach in simulation scenarios (see Materials and Methods), which cover a range of expected situations in HDX studies. We perform simulations with four or five time points and two or three replicates.

**Figure 1:**
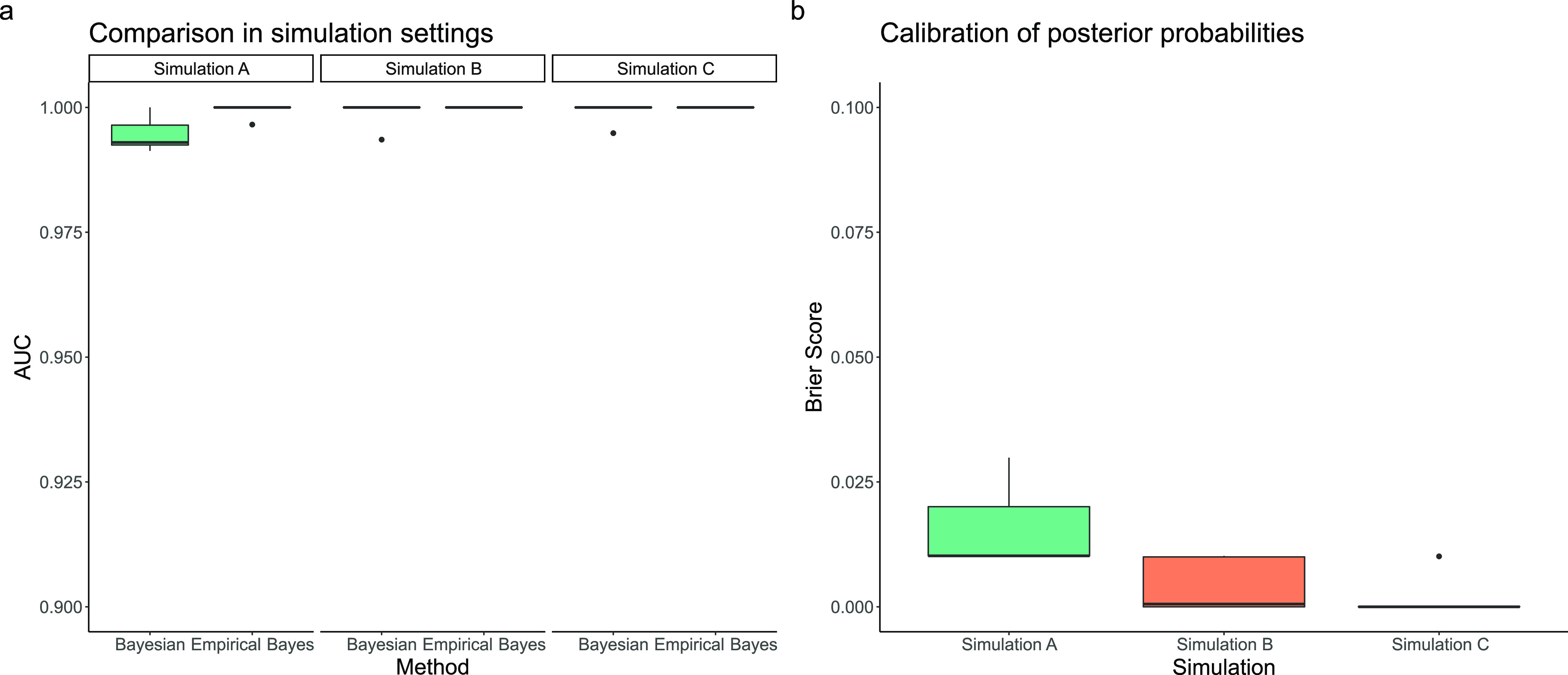
Simulation study to compare statistical methods on HDX-MS data. (a) Area-under-the-curve (AUC) for both the proposed Bayesian approach and the empirical Bayesian approach. The effective difference is too small to suggest any practical difference between the approaches. (b) Brier scores for our Bayesian approach. Values are close to 0 suggesting high quality calibration. See Materials and Methods for details on simulations; briefly, simulation A has four time points and three replicates. Simulation B has four time points and two replicates. Simulation C has five time points and two replicates.

We examine two quantities while performing these simulations. The first is to compare performance to our previously proposed empirical Bayes method for HDX-MS data^12^ (which was shown to be the most powerful method for determining deuterium differences in HDX-MS data). Here, we use the area-under-the-curve (AUC) of the TPR (true positive rate)–FPR (false positive rate) curve. Values close to 1 indicate perfect performance, while values of 0.5 suggest random guessing. We ran five simulations in each setting and reported the distribution. We find that both methods give AUC values close to 1 (see Figure 1). The minor differences between the two methods are too small to be relevant in practice.

In addition, we compute the Brier score (see methods) for our Bayesian approach. The Brier score assesses whether the posterior probabilities are correctly calibrated. This means that if there is a perturbation to the HDX kinetics, we expect probabilities close to 1 and if there is no perturbation, we expect probabilities close to 0. The Brier score quantifies the average calibration-error. As can be seen from Figure 1 the errors are, on average, less than 1 percentage point. This means that if there are no perturbations to the HDX-MS kinetics our approach could report a probability of 0.01 of a perturbation, and if there is a perturbation our approach could report a probability of 0.99 of a perturbation. This represents almost perfect calibration, and our probabilities can be interpreted as forecasts. Given that our Bayesian approach has a statistical performance similar to that of the empirical Bayes approach, the remainder of the paper focuses on demonstrating the interpretation advantages of our Bayesian method. In the Supporting Information, we consider selecting the best model for HDX data depending on the type of exchange kinetics, using structural spike-in data.^17^

### 3.3 Controlling False Positives: An MBP Case-Study

To demonstrate that our Bayesian analysis controls false discoveries, we perform a permutation experiment, using an experiment on Maltose-Binding protein (MBP) generated in seven replicates across four HDX labeling times.^17^ Additional experiments were carried out in triplicate for the W169G (tryptophan residue 169 to glycine) structural variant. Here MBP-W169G was spiked into the wild-type MBP sample in 5, 10, 15, 20, and 25% proportions, and a further experiment included a 100% mutant sample. All data were analyzed on an Agilent 6530 Q-TOF mass spectrometer, and raw spectra were processed in HDExaminer. The seven MBP samples without any structural variant can be used as a null experiment by partitioning the replicates falsely into two conditions. That is, three of the samples are labeled condition *A* and four samples are labeled condition *B*, arbitrarily. We randomly permute the samples labeled *A* and *B*, five times. We then computed the posterior probability that each peptide is perturbed (alternative model). For each permutation this is visualized in a histogram of all the probabilities (see Supplementary Figure S5). We see that the posterior probability is never above 0.05 suggesting excellent control of error rates; that is, we never give confident support to the wrong model. We also check that the probabilities are calibrated by computing the Brier score for each permutation experiment. We plot the Brier scores as a boxplot and see that they are essentially 0, indicating good calibration (see Supplementary Figure S5). The analysis of the structural spike-in experiment is in the Supporting Information.

### 3.4 Case-Study: Epitope Mapping of HOIP-RBR

Ubiquitination is a key post-translational modification and acts as a molecular toggle in the regulation of cellular processes. HOIP is a member of the E3 complex LUBAC and plays a significant role in immune signaling, by conjugating linear polyubiquitin chains.^41−43^ The active domain of the LUBAC complex is located in the HOIP-RBR domain, and inhibition of LUBAC activity has been shown to be of potential therapeutic benefit. To support structure-based inhibitor design, Tsai et al.^18^ sought to develop single chain antibody-based crystallization chaperones. As part of their study, they performed epitope mapping of a library of synthetic domain antibodies (dAbs) using HDX-MS. To identify binding epitopes, they looked for “protection” signatures, that is, surface amides that incorporate deuterium more slowly in the bound state as they are shielded from the solvent. Tsai et al.^18^ performed HDX-MS experiments for HOIP-RBR upon single domain antibody (dAb) complexation and in the APO state. Mass spectrometry was performed using a Waters Synapt G2-Si instrument, and raw data was processed using DynamX. HDX-MS measurements were taken at 0, 30, and 300 s post exposure to heavy water, for 13 dAbs at different molar concentrations. Only a single replicate measurement was taken in each state so that measurements of many different dAbs could be made.^44,45^ This means that statistically rigorous analysis is challenging and only obvious binding epitopes could be identified.^12,18^ Here, we apply our Bayesian model to two of the dAbs with the hope of quantifying our confidence in the binding epitope and the possible conformational changes of HOIP upon dAb-complexation.

### 3.5 HDX-MS Analysis of HOIP-RBR-dAb25

First, we focus on dAb25, one of 13 dAbs, that demonstrated nonstandard HDX behavior for this complex.^12,18^ By visual examination, Tsai et al.^18^ noted deprotection signatures and, thus, hypothesized that dAb25 locks HOIP-RBR in a more open conformation. However, this qualitative approach is prone to bias and errors. They could not apply a quantitative approach such as the *t* test or a linear mixed model because neither of these was applicable to the data due to lack of replication. Recently, an empirical Bayes function model was able to identify statistical signficant differences.^12^ That analysis identified eight peptides for which the deuterium kinetic were altered (adjusted *p*-value < 0.05), suggesting that the binding epitope was located in the in-between ring (IBR) of HOIP (see Supplementary Figure S9). However, that approach required the fixing of the parameters in a Weibull model to *b* = 0.5, *p* = 1, and *d* = 0. This reduces the flexibility of the model and hence kinetics that deviate from these assumptions will be poorly modeled. Our Bayesian approach allows us to regularize rather than fix these parameters facilitating modeling of a larger range of kinetics. In particular, the rate constant *b* can be inferred rather than fixed. Our proposed Bayesian logistic model is fitted using Markov-chain Monte Carlo (MCMC), and so uncertainty in these parameters is propagated to the uncertainty in the underlying time-dependent function.

**Figure 2:**
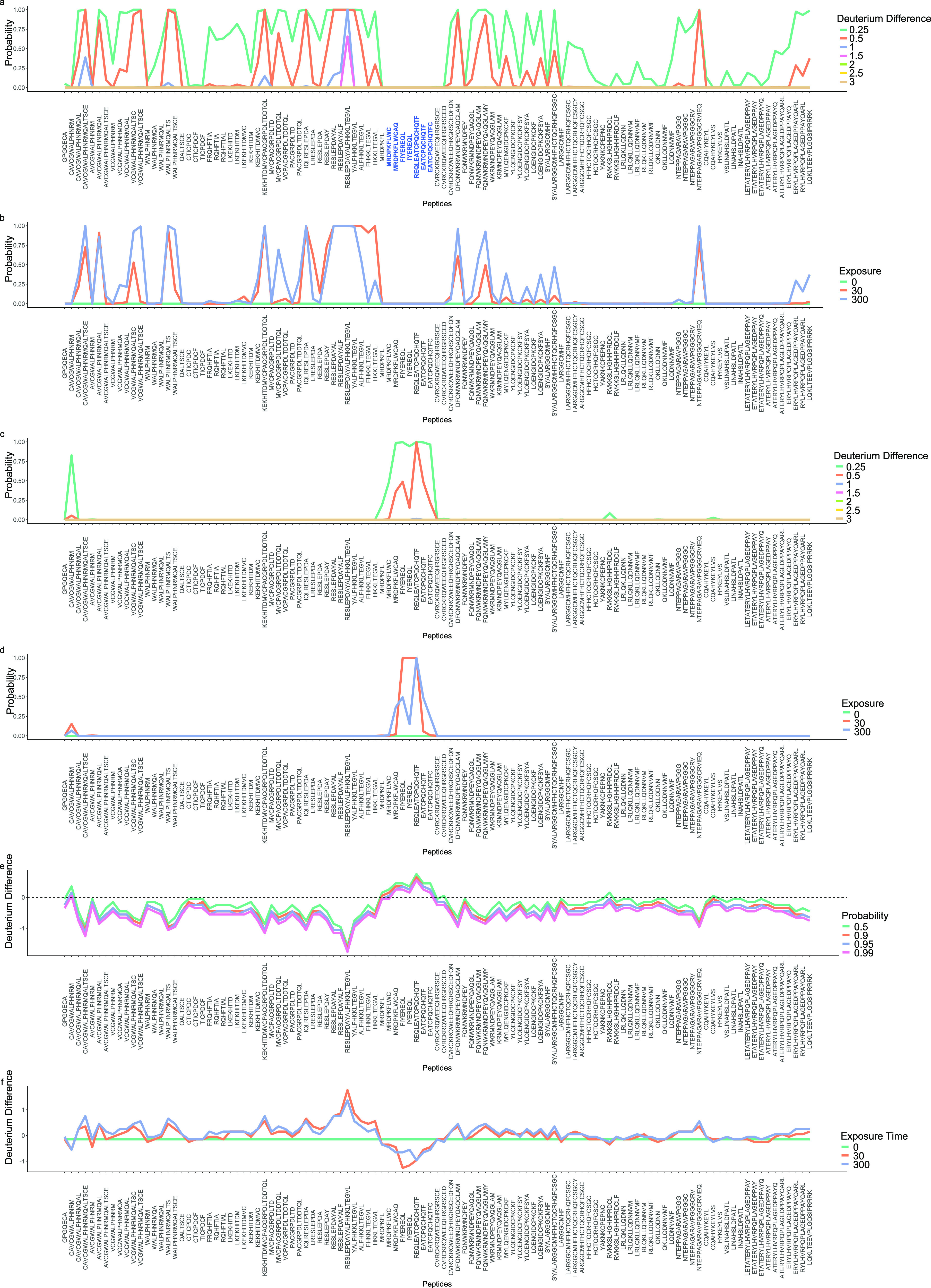
Posterior quantities of interest from Bayesian analysis. (a) The probability that the deuterium difference is higher than a particular effect size in the dAb-bound state; in general, we see a tendency for the dAb-bound state to incorporate more deuterium. There is a clear band of overlapping peptides (highlighted) where there is no deprotection. (b) Consistency of probabilities across the temporal dimension of the data. Plotted is the probability that the deuterium difference is higher than 0.5 in the dAb-bound state. (c) The probability that the deuterium difference is greater in the apo state (epitope mapping) for a number of effect sizes. An epitope is clearly visible. (d) Consistency of probabilities across the temporal dimension of the data. Plotted is the probability that the deuterium difference is greater in the apo state. An identified epitope is consistent across the temporal dimension. (e) We plot the largest value *d* such that *P*(Δ*D* > *d*) = *p* for different values of *p*, and the dAb-bound state is used as the reference. We see that in general a tendency of increase incorporation in the dAb-bound state and the epitope is clearly identifiable (cluster of values above 0). (f) Consistency across the temporal dimension of the data. We plot the largest value *d* such that *P*(Δ*D* > *d*) = *p* for different values of *p*, the apo state is used as the reference. We see a general tendency for the increased incorporation of deuterium in the dAb-bound state. The epitope is identifiable from a cluster of values below 0.

Our Bayesian approach facilitates a number of probabilistic computations. First, we examine cases where there is increased deuterium exchange as a result of antibody binding. That is, regions of HOIP-RBR that are more solvent accessible. For this, we compute the *P*(Δdeuterium_(dAb_25_,APO)_ > *d*) for fixed values of *d*, where we use Δdeuterium_(*A*,*B*)_ to denote the deuterium difference between states *A* and *B*. This computation was made at 300 s post exposure to heavy water with time dependent computations made later. Figure 2a shows a plot of this probability as a function of peptide, arranged in the protein order. First, we observe that peptides generally appear to increase their deuterium incorporation when the antibody is bound, suggesting that this antibody holds HOIP-RBR in a more open conformation. Second, we note that these effect sizes are generally small/moderate, suggesting that these changes are likely subtle. Finally, we observe a clear region in the center of the plot where deuterium deprotection is not observed, a region that could be an epitope on further inspection. To examine whether these kinetics are consistent with respect to the time dimension of the HDX measurements, we can compute *P*(Δdeuterium_(dAb_25_,APO)_ > *d*) for a fixed *d* = 0.5 for each time point. Figure 2b plots these quantities for each peptide. This plot supports the same conclusions as in Figure 2a.

To identify the epitope, we switch the ordering in our computation *P*(Δdeuterium_(APO,dAb_25_)_ > *d*). We compute the relevant quantities again and plot in Figure 2c,d. Here, the probable epitope is evident from a cluster of several peptides in the center of the figures. This includes a cluster of seven peptides with some probability of a reduced level of deuterium exchange in the dAb-bound state. This more than triples the previous evidence for an epitope in this location given by the empirical Bayes approach.^12^ This suggests that we confidently identified the epitope rather than spurious measurement fluctuations. Since our approach is probabilistic, these probabilities could be used alongside crystal structure data or machine learning based approaches as complementary evidence.

More elaborate probabilistic computations are possible. For example, we can consider the equation *P*(Δdeuterium_(APO,dAb_25_)_ > *d*) = *p* for some *p*, say 0.99. We note that if *d*_1_ > *d*_2_ then *P*(Δdeuterium_(APO,dAb_25_)_ > *d*_1_) ≤ *P*(Δdeuterium_(APO,dAb_25_)_ > *d*_2_) because the integration is over a larger set. Thus, decreasing the value of *d* cannot decrease *p*. From here, we can find the largest value *d* such that *P*(Δdeuterium_(APO,dAb_25_)_ > *d*) = *p*. We plot this quantity for different values of *p* in Figure 2e. Of course, if the largest such value of *d* is less than zero, this suggests that deuterium is being incorporated in those peptides in the antibody bound state. This procedure allows us to clearly identify the threshold at which peptides become interesting and facilitates the identification of the peptide with the strongest evidence for differences. This plot further highlights the probable binding epitope. For illustration, we include the temporal version of this plot in Figure 2f, now taking the apo state as the reference and letting *p* = 0.95.

Finally, we examine a peptide in the RING1 region of HOIP-RBR (MVCPACGRPDLTDDTQL [742–758], see Figure S10b), which has an increase in the level of deuterium incorporation upon dAb25 complexation. We can visualize a number of posterior quantities from this analysis, including the posterior predictive distribution of the entire time-resolved kinetics (Figure S10); the posterior predictive distribution, as a violin plot, of deuterium incorporation at specific times (Figure S10) and the posterior predictive distribution, as a violin plot, of the deuterium difference at specific times (Figure S10). These plots allow us to see that a deuterium difference is already evident by 30 s but is much more pronounced at 300 s. We are able to report probabilities and hence confidence in our conclusions, allowing us to weigh up HDX-MS data in the context of other experiments. In summary, our Bayesian approach allows powerful visualization and computation for HDX data and increases our ability to carefully draw quantitative insights from our data.

### 3.6 HDX-MS Analysis of HOIP-RBR-dAb3

**Figure 3:**
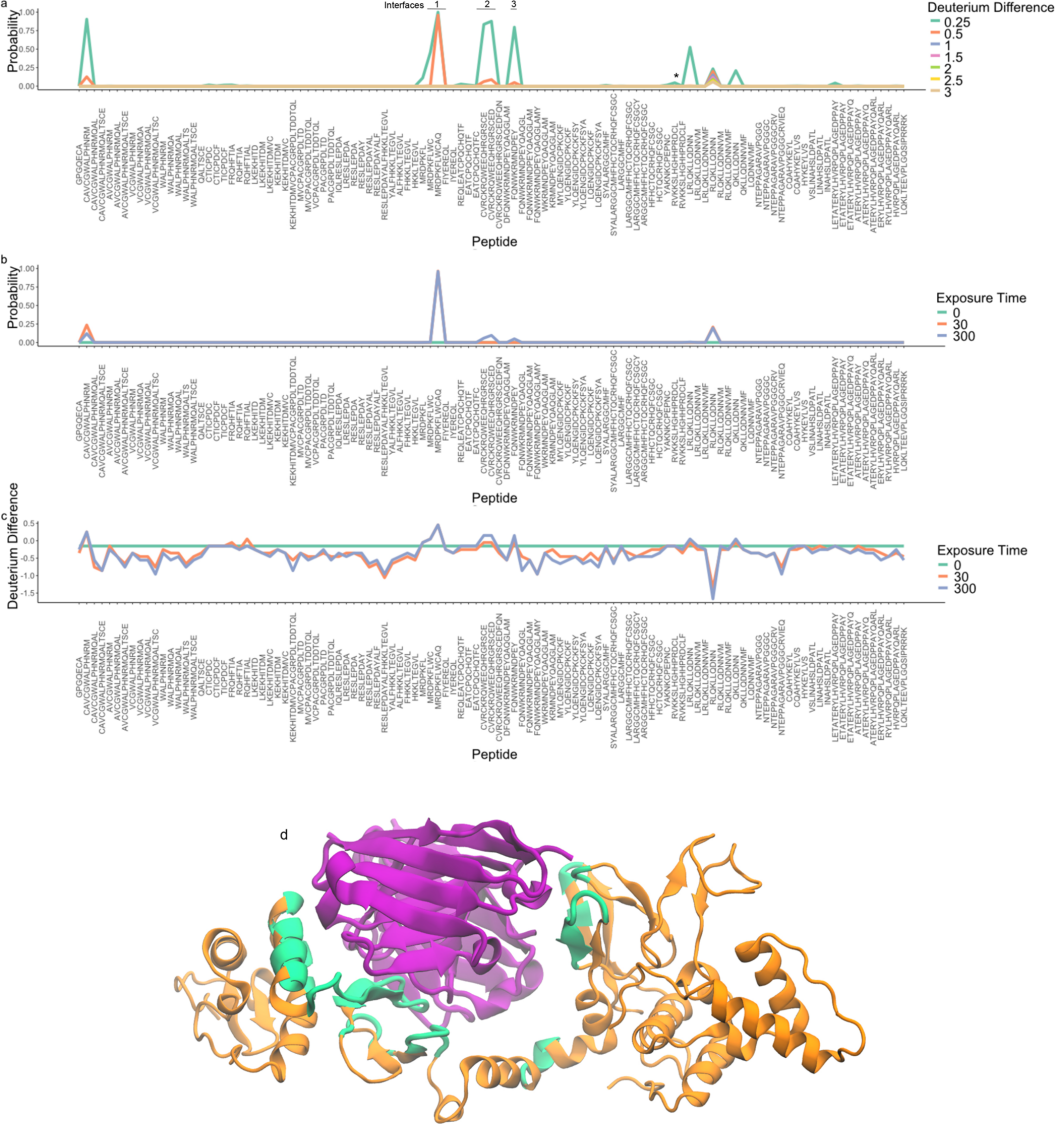
Posterior quantities to identify the epitope in HOIP-RBR-dAb3. (a) Probability that the deuterium incorporation is reduced by a particular effect size in the dAb bound state. (b) The temporal consistency of the computation in panel a with *d* = 0.5. (c) The largest value of *d* such that *P*(Δ*D* > *d*) = 0.95 for different time points plotted as a function of the peptide. (d) Cartoon representation of HOIP-RBR-dAB3. Distance analysis of cocomplex of HOIP-RBR-dAB3. Residues within 8 Å of the dAb3 dimer (violet) are highlighted in green on HOIP-RBR (orange) PDB: 6SC6.

We now turn to another epitope mapping task from the HOIP-RBR data set. Tsai et al.^18^ report an X-ray crystal structure of HOIP-RBR-dAB3, where dAb3 forms a dimer (see Figure 3 d). Tsai et al.^18^ show that the dAb3 dimer contacts the IBR domain of HOIP from residues on the complementarity determining regions (CDRs). Residues on each monomer form hydrogen bonds with two distinct clusters of residue on HOIP, suggesting that the dAb3 dimer has interactions with different sections of the IBR. However, no previous analysis of HDX-MS data for this system could identify any binding epitopes. More specifically, application of the empirical Bayes functional method led to no peptides being identified as having significant changes in deuterium incorporation in this experiment.^12^ Hence, we apply our Bayesian approach to determine whether it could identify these interactions from the HDX-MS data.

Figure 3 plots the relevant probabilistic calculations for epitope mapping that we previously outlined for HOIP-RBR-dAb25. From visual inspection of these plots, we identify peptides indicative of an epitope. These three peptides include residues that were identified interacting with dAb3 in the crystal structure: arginine (R792) and aspartic acid (D793).^18^ These residues interact with residues on the CDR2 region of the first dAb3 monomer, confirming that HDX-MS could localize the first binding interface in agreement with the crystal structure. A second small cluster of two peptides shows evidence of small deuterium differences. These two peptides include an arginine (R827) and a lysine (K829) that were also identified to interact with the CDR3 region of the second dAb3 monomer. Again, this demonstrated concordance between our Bayesian analysis of HDX-MS data and the crystal structure. A third potential, but less probable, location for an epitope is in peptide FQNWKRMNDPEY [844–855] (see Supplementary Figure S9c), though it is not supported by overlapping peptides. Performing distance analysis on the cocrystal structure of HOIP-RBR-dAb3 (PDB: 6SC6), we find 3 interfaces within 8 Å of the dAb3 dimer (Figure 3d). This includes a segment between D766 and E809, another segment between C817 and W832, and finally between R849 and Q858. These three interfaces are concodant with HDX findings and suggest that Bayesian analysis combined with HDX is able to sublocalize interaction sites.

Other peptides that appear to have some changes to their deuterium incorporation are either at low probability or are inconsistent with overlapping peptides, suggesting these are more likely random fluctuations rather than possible binding epitopes. This shows that bespoke statistical approaches for HDX-MS data can provide complementary and consistent evidence to other structural data, which was not possible with other statistical methods.

Next, we performed a differential solvent accessibility analysis for HOIP-RBR-dAb3. Briefly, we computed the accessible solvent area (ASA) for each residue of the unbound and bound forms of HOIP-RBR.^46^ We identified two residues with confident differences (see the Methods and Materials as well as Supplementary Figure S8). The first is residue D793, which is contained within confident peptides displaying signatures of solvent occlusion. The second is residue R928, which is distal from the hypothesized interaction sites. The relevant peptide from HDX-MS analysis is RVKKSLHGHHPRDCL [917–931] (see Supplementary Figure S9d), for which we observed a small but nonzero probability in the perturbation of its HDX kinetics. Examining the crystal structure, we see a rearrangement of the turn on which this residue is localized: ψ_[927,928,929]_ = (173, −35, −37) in unbound form and in bound form ψ_[927,928,929]_ = (162, −18, 16). The apparent contradiction with HDX-MS analysis, which suggests a small difference, and the large changes observed in the crystal structure arises for two reasons. The first is the signal for HDX-MS analysis is averaged over the residues of the measured peptide. Second, the crystal structure is only a single conformation of HOIP-RBR-dAb3, while the HDX-MS analysis is again averaged over the ensemble of possible conformations. This highlights the utility of HDX-MS to report on the ensemble of structures and the ability of Bayesian analysis to identify subtle changes. Supplementary Table S1 in the Supporting Information summarizes the agreement of the cocrystal structure and the HDX-MS data.

We further explored the 3 peptides that overlapped with R792 and D793, so that we could carefully examine their kinetics. Time-resolved kinetic plots and violin plots (Supplementary Figure S11) show that the posterior predictive distributions overlap for peptides MRDPKFL and MRDPKFLWC, however there is a clear suggestion that antibody binding is reducing deuterium incorporation. Furthermore, the posterior predictive distribution for MRDPKFLWCAQ shows much clearer evidence for a reduction in deuterium incorporation, suggesting residues C799-A801 are likely important for this interaction. Examining the cocrystal structure of the dAb3 dimer and HOIP-RBR, we see that the loop containing C799 and A800 are within 8 Å of the first dAb3 monomer and so potentially interacting (Figure 3d). We then carefully examined HOIP-RBR-dAb3 (bound HOIP) and HOIP in complex with the ubiquitin-transfer complex (free HOIP), zooming in on residues C799 and A800 (Supplementary Figure S11). In bound HOIP, we see that the backbone nitrogen of C799 forms a clear hydrogen bond with the carbonyl from residue F804, and the nearby zinc cation suggests that this loop is structured (Supplementary Figure S11j)). Meanwhile, in unbound HOIP, we observe the same residue contacts. Hence, the proximity of the antibody likely stabilizes this loop and reduces HDX at C799. In contrast, in both bound and unbound HOIP the amide of A800 is likely bound to water, suggesting that it can freely exchange, with no hydrogen bond directly formed with the antibody dimer. This suggests that C799 is a critical residue for this interaction. Hence, the results of our Bayesian analysis on HDX-MS data are consistent with the X-ray crystal structure of HOIP-RBR-dAb3 and that complementary information can sublocalize binding epitopes.

### 3.7 Case-Study: HDX-MS Analysis of BRD4 with Bromodomain Inhibitor I-BET151

**Figure 4:**
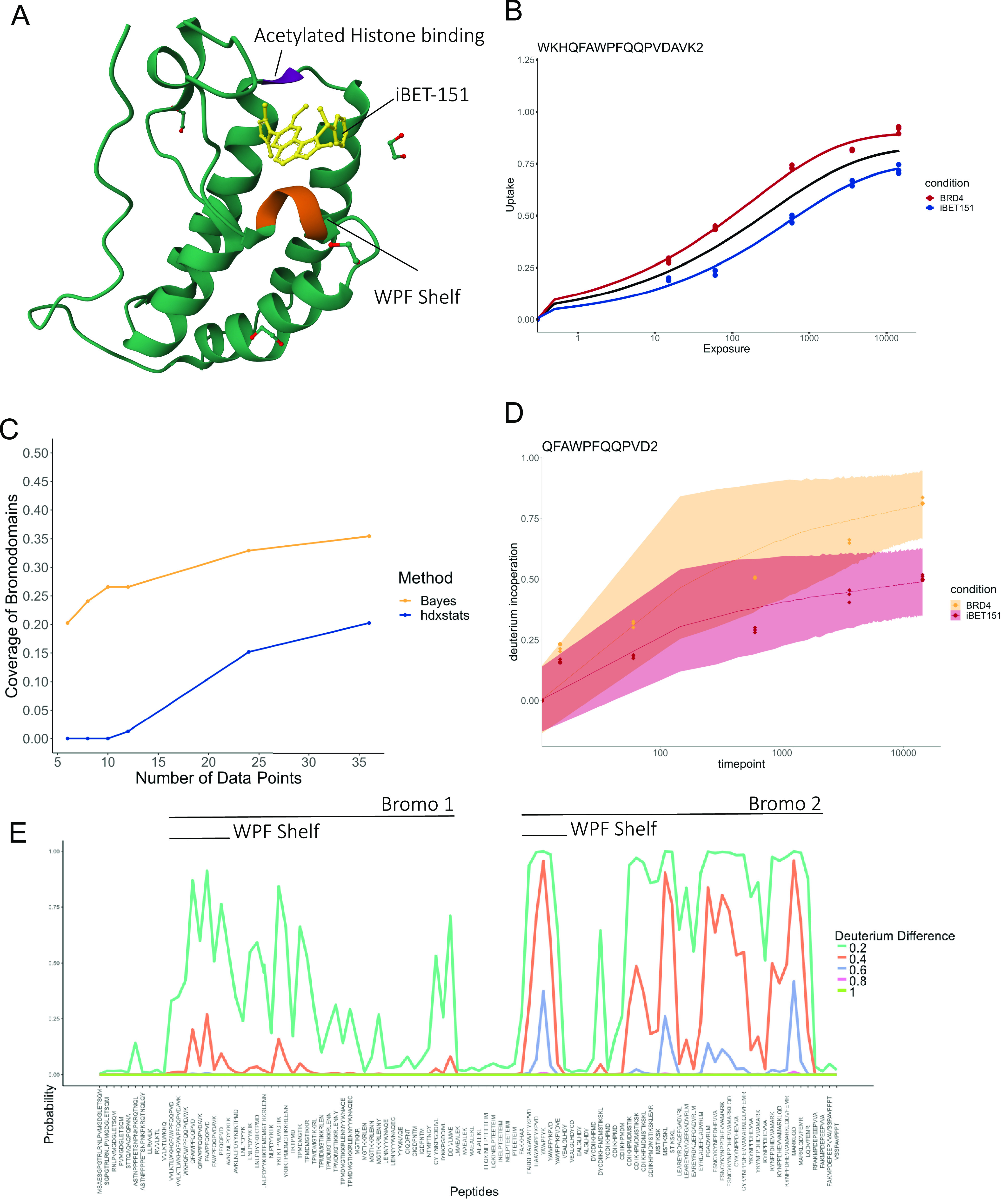
HDX-MS analysis of BRD4. (a) The structure of Bromodomain 1 of BRD4 in complex with I-BET151 (PDB: 3ZYU). (b) Examle kinetic plots of peptide covering the WPF shelf showing a clear protection signature. (c) Data ablation results comparing the empirical Bayesian approach (hdxstats) with the Bayesian approach developed here (Bayes) (d) Example kinetic plots from the Bayesian analysis fitted to three data points from each protein state (condition). Fitted data are plotted in circles, while data from the full experiment, which are not seen by the method, are overlain by diamond pointers. (e) Probability estimates of protection for the different peptides of BRD4. Deuterium differences are shown by different colors.

Bromodomains (BDs) are evolutionary and structurally conserved regions of Bromodomain-containing proteins (BCPs) that bind acetyl-lysine. BCPs are known regulators of gene-transcription and chromatin remodelling, thus are potential therapeutic targets.^47^ Bromodomains contain a common folding pattern of four antiparallel alpha-helices oriented in a left-handed twist (see Figure 4A)). The acetyl-lysine binding site is located at the rear of the helical bundle that contains a conserved asparagine and WPF shelf—known essential residues for recognition of the PTM. The specificity and affinity of cognate peptides is governed by the loops that flank this pocket. We refer to Chung and Tough^48^ for a review. The wide therapeutic opportunity for bromodomain inhibitors motivates the desire for rapid and sensitive structural profiling of these inhibitors on their targets.^49^

BRD4 is a member of the bromodomain and extra terminal (BET) family of proteins, and is a desirable drug target because of its conserved tandem bromodomain and its role in cancer epigenetic.^50^ BRD4 is inhibited by the selective small molecule GSK1210151A (I-BET151), which provides an effective treatment for MLL-fusion leukemia.^51^ Here, we performed HDX-MS of BRD4 with and without bound I-BET151 to gain a further understanding of the molecular details of the interaction. Since we have prior knowledge that I-BET151 binds at the bromodomains, we can assess the sensitivity of our proposed Bayesian approach against our previous empirical Bayes approach (“hdxstats”).^12^ HDX-MS was performed across six time points (0, 15, 60, 600, 3600, 14 400 s) for three replicates, and BRD4 was enzymatically digested online using Nepenthesin-2 (Affipro). Mass spectrometry was performed using a Waters Synapt G2-Si instrument, raw data was processed using HDExaminer and data was normalized using a fully deuterated control. We refer to the Methods and Materials for additional details.

We first analyzed the complete data set with our previous empirical Bayes approach and identified clear stabilization at the WPF shelfs and in the wider Bromodomain regions. An example protection signature is given for a WPF containing peptide in Figure 4B. We found that almost 20% of peptides contained in the Bromodomains demonstrated significant levels of protection (*p* < 0.01 and reduction in residual variance by 0.1). However, application of the Bayesian approach proposed here revealed 35% of peptides in the Bromodomains with protection signatures (probability that deuterium incorporation decreased by 0.2 was at least 0.99).

While it is known that the binding of I-BET151 is at the WPF shelf, this region does not always display the strongest stabilization signature; rather, the whole bromodomain is stabilized. We measure sensitivity as the proportion of bromodomain peptides that can be confidently determined as stabilized. It is a limitation of HDX-MS that we cannot differentiate a true binding site from other changes that reduce HDX. In order to evaluate whether the additional sensitivity of our Bayesian approach would allow us to reduce the amount of data profiled, we sequentially removed data from the experiment and determined how many peptides covering the bromodomains were identified with high confidence stabilization patterns. Since we do not know a priori which time-points would display a difference, we maintain all time points before adding replicated data. Hence, 10 data points is 1 replicate with 5 data points in each condition. As expected, both methods’ performance degraded as data points were removed (see Figure 4C). However, our proposed Bayesian approach suffered to a much lesser degree and provided a similar performance, with only six data points, to that achieved by the empirical Bayesian approach using the full data set. Non-Bayesian and empirical Bayesian approaches will always suffer the issue that if fewer data points are measured than parameters in the model, then it cannot determine any significance. Given there are two states each requiring a 4 parameter model, a minimum of 9 data points are needed. However, even using 10 data points by removing the 600 s data point for both conditions, we found that the smallest *p*-value was for a WPF-shelf peptide FAWPFQQPVD at just above 0.1—well above a typical 0.01 threshold. This is not an issue for our proposed fully Bayesian approach. Hence, its improved sensitivity suggests that our approach could aid rapid profiling of the mode of action of large libraries of small molecule inhibitors using HDX-MS.

We then examined our Bayesian approach using only three data points under each condition (BRD4 ± I-BET151). In Figure 4D, we plot an exemplary set of model fits. The clear protection signature for this WPF covering peptide is still evident despite the wide uncertainty bands. Of course, with only 3 data points (0, 15, 14 400 s) and no replicates, the fits are poor with high uncertainty but this is the intention of this analysis. We demonstrate that even with low data, deuterium differences can be detected albeit with low confidence. Overlaying the full data for this peptide in diamond-shaped pointers demonstrates that these uncertainty bands are faithful and are conservative in their estimation of confidence. This is reassuring, as we do not wish to inflate false positives by underestimating the uncertainty. Figure 4E shows global probability estimates of protection for the complete set of profiled peptides. We see widespread stabilization of BRD4 in complex with IBET-151 and, perhaps surprisingly, a more pronounced protection pattern for the second Bromodomain. Examining the raw uptake profiles, we suggest that this protection pattern is explained by the second Bromodomain being more flexible than the first rather than stronger binding of I-BET151. Supplementary Figure S12 shows this by generally more rapid deuterium incorporation for peptides covering the second Bromodomain than for the first. Hence, these results demonstrate that our Bayesian approach is a sensitive approach for the analysis of the mode of action of small molecule inhibitors using HDX-MS.

## 4 Discussion

Current statistical approaches applied to or developed for HDX-MS data lack flexibility. For example, *t* tests and linear mixed models are applicable only with sufficiently large numbers of replicates. A recently proposed empirical Bayes approach was able to identify difference when the *t* test and linear mixed models where not applicable.^12^ However, that approach was still unable to identify differences in some HDX-MS experiments.^12^ To overcome these limitations, we developed a Bayesian approach to modeling HDX-MS data. Our approach combines a likelihood model that captures the time-dependent kinetics of HDX data, either as a logistic model or a Weibull model. We place priors on the parameters of these likelihood models, which rules-out unrealistic parameter values and allows us to shrink residuals toward zero. We sample from the posterior distribution of this model using a variant of Hamiltonian Monte Carlo (HMC).^35,52^ This posterior distribution allows us to quantify uncertainty in the parameters of our model or, more generally, any posterior quantity that we are interested in.^13^

Through careful simulations, we show that our Bayesian model correctly identifies true perturbations and has a similarly high performance when compared to the previous empirical Bayes approach. We also demonstrate that our model is correctly calibrated by examining the Brier score.^37^ We then use simulations and a structural spike-in experiment to demonstrate the power of Bayesian model selection.^19^ Here, we use the Bayesian tool-kit to demonstrate that EX1 kinetics are modeled better by the Weibull model compared with a logistic model. Furthermore, we rule out a more complex random plateau model. Then, we showed that our Bayesian approach does not unnecessarily inflate false positives.

We then turn to an epitope mapping experiment for HOIP-RBR, an E3 ubiquitin ligase, involved in immune signaling.^41^ We first consider HOIP-RBR-dAb25, to demonstrate a number of new computations and visualization. Our analysis suggests that a region of the IBR contains the binding epitope, by identifying 7 key peptides in this region with reduced HDX. This more than triples the evidence for an epitope in this region compared with previous analysis.^12,18^ Our analysis also reaffirms the idea that this antibody holds HOIP-RBR in a more open conformation, as most peptides have a tendency to increase their deuterium incorporation into the bound state. We proceed to consider HOIP-RBR-dAb3 because previously it has not been possible to identify an epitope from this HDX data.^12,18^ However, our Bayesian analysis suggests three possible interfaces on the IBR that could be the binding epitope. All these regions are consistent with distance analysis on the cocrystal structure of HOIP-RBR-dAb3. Using this crystal structure, we sought to further interrogate the HDX kinetics. We suggest that the dAb3 dimer likely stabilizes the interaction network of residue C799. Our findings demonstrate that a Bayesian analysis can identify important binding epitopes from HDX-MS data that could not be found with other approaches. This methodology presents a powerful alternative approach to the statistical analysis of HDX-MS, allowing us to quantify the uncertainties in our analysis. Finally, we compare our previous approach with our Bayesian analysis on an application to BRD4 in complex with selective small molecule I-BET151 and demonstrate that our approach can significantly reduce the data required for small molecule mode of action studies.

## Data Availability

Data to reproduce the figures is provided in the Supporting Information. Experimental data are available from the original papers. The BRD4 data is provided in the Supporting Information. Monte Carlo data is available from (10.5281/zenodo.6855078). Stan files implementing the methods are provided as part of the Supporting Information

## Supporting Information Available

Supplementary Code: Stan code for Bayesian models (ZIP)
Supplementary Figure S1: Example prior predictive checks. Supplementary Figure S2: Example posterior predictive checks. Supplementary Figure S3: Example trace plots. Supplementary Figure S4: Example trace plots. Supplementary Figure S5: Bayesian analysis control error rates in null experiments. Supplementary Figure S6: Examining model selection for EX1 kinetics. Supplementary Figure S7: Examining model selection for structural spike-in kinetics. Supplementary Figure S8: Differential solvent accessibility analysis. Supplementary Figure S9: HOIP-RBR structures. Supplementary Figure S10: Posterior kinetics for peptide from the Bayesian analysis. Supplementary Figure S11: Posterior predictive kinetic plots for three peptides. Supplementary Figure S12: BRD4 relative deuterium uptake in Bromodomains. Supplementary Table S1: A summary of the correspondence between the Bayesian analysis of HDX-MS data and cocrystal structure (PDF)
Supplementary Data set: BRD4 HDX-MS data (ZIP)
