# Supplementary material for: A Functional Bayesian Model for Hydrogen–Deuterium Exchange Mass Spectrometry
Source: J Proteome Res. 2023 Aug 15;22(9):2959–72. doi: 10.1021/acs.jproteome.3c00297 (PMC10476270; doi:10.1021/acs.jproteome.3c00297)
Supplement: Supplementary file 2 — pr3c00297_si_002.pdf [file pr3c00297_si_002.pdf]

Supplementary material: A functional Bayesian model for hydrogen-deuterium exchange mass-spectrometry

Oliver M. Crook * [1], Nathan Gittens[2], Chun-wa Chung[2], and Charlotte M. Deane[1]

[1] *Department of Statistics, University of Oxford, Oxford, UK*
[2] *Structural and Biophysical Sciences, GlaxoSmithKline R&D, Stevenage, UK*

1 Detailed methods

1.1 Prior and posterior predictive checks

* oliver.crook@stats.ox.ac.uk

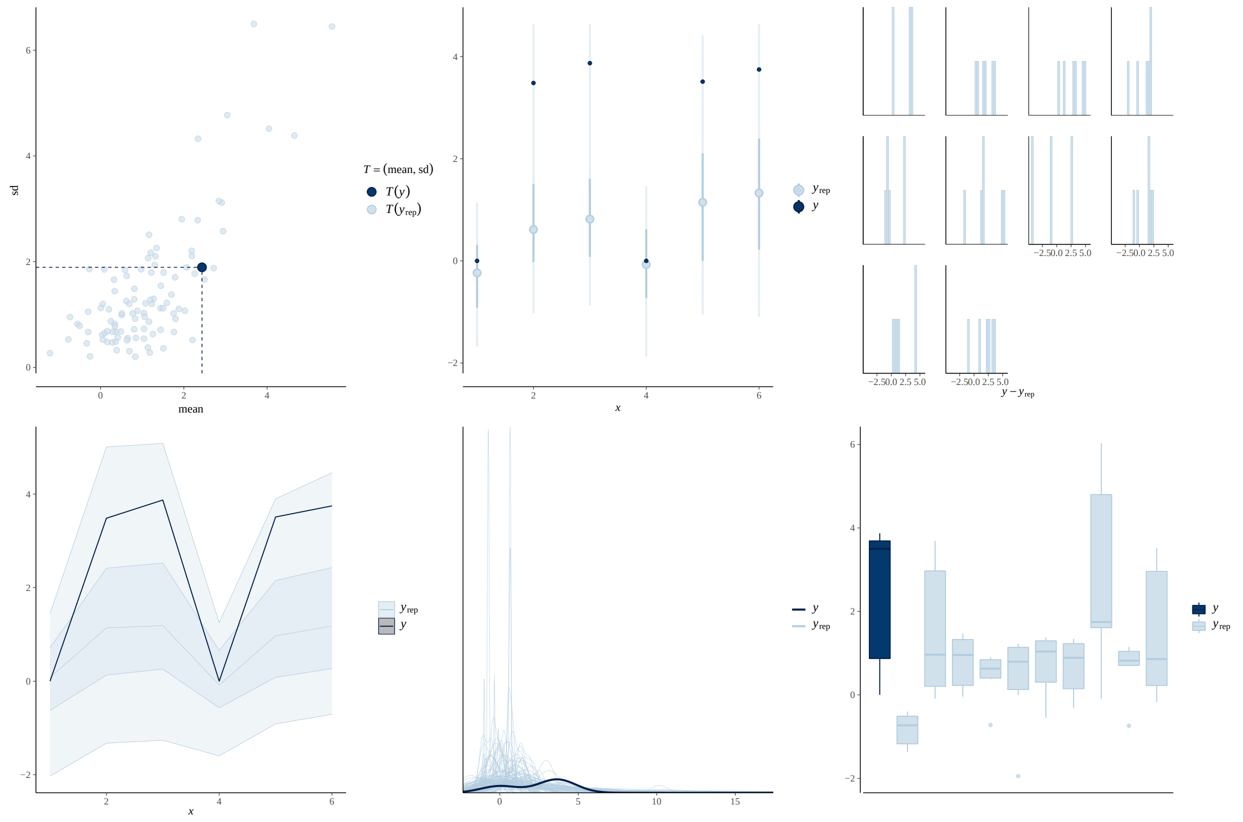

Supplementary Figure S1: **Example prior predictive checks.** Each panel shows a prior predictive check for a different summary statistics. We simulate from the prior predictive distribution denoted as y_{rep} and compare to the observed data y. We see that the prior predictive is diffuse but correctly located

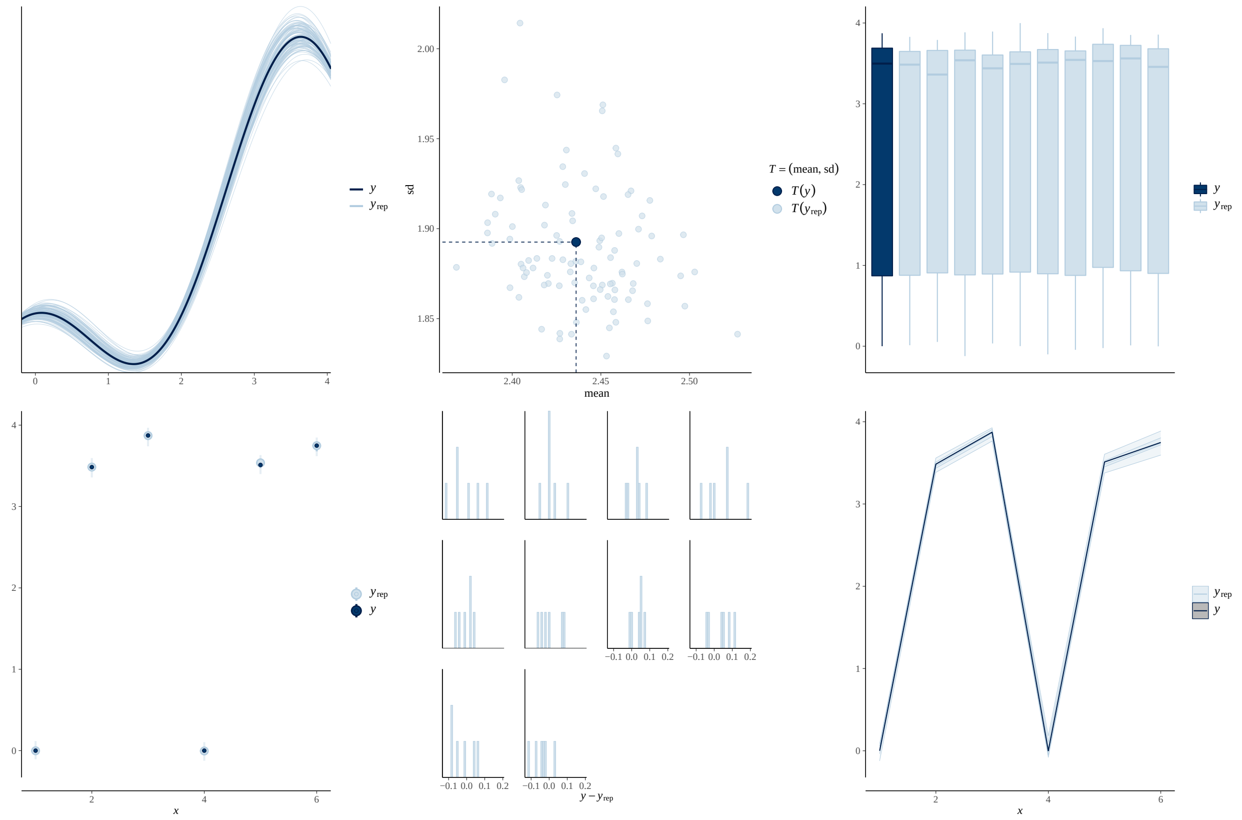

Supplementary Figure S2: **Example posterior predictive checks.** Each panel shows a posterior predictive check for a different summary statistics. We simulate from the posterior predictive distribution denoted as y_{rep} and compare to the observed data y. We see that the posterior predictive is concentrated and correctly located

1.2 Convergence analysis

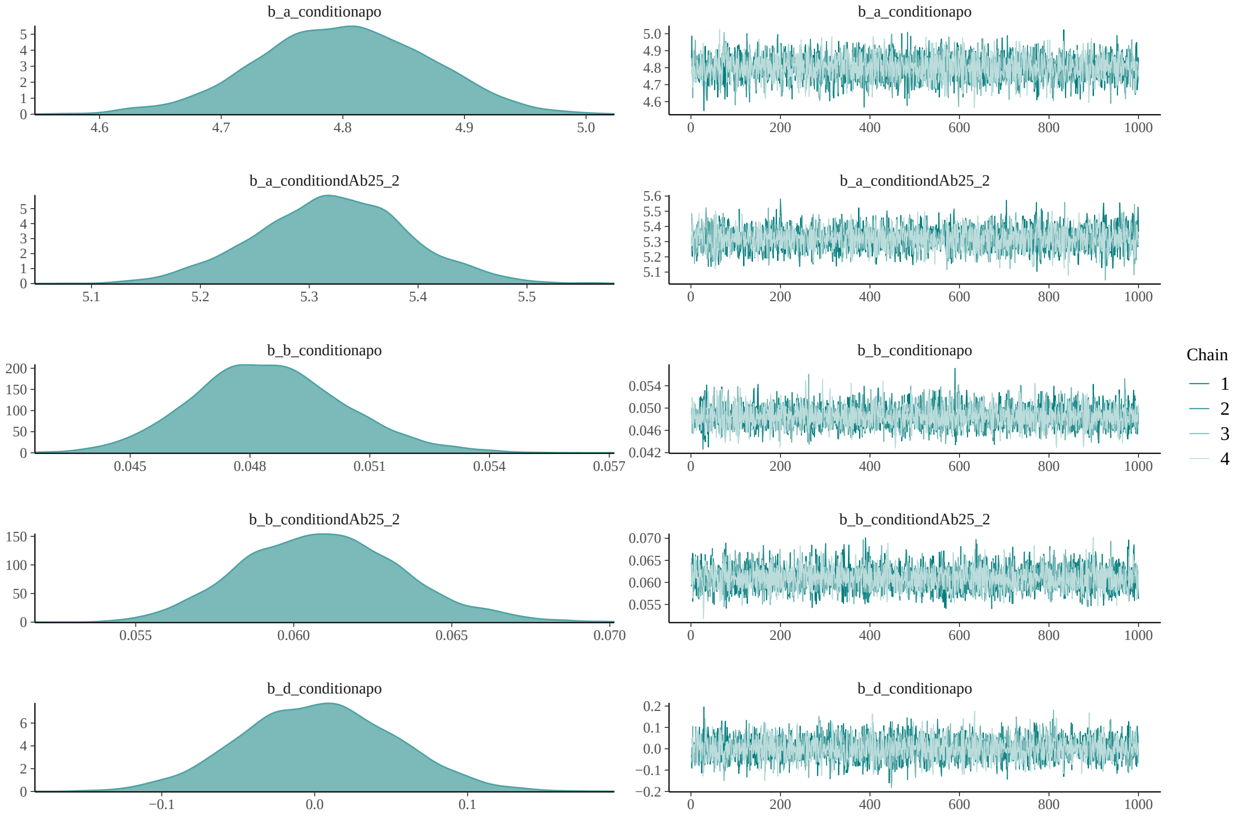

Supplementary Figure S3: **Example trace plots.** Trace plots for example parameters from MCMC runs, convergence is clearly achieved

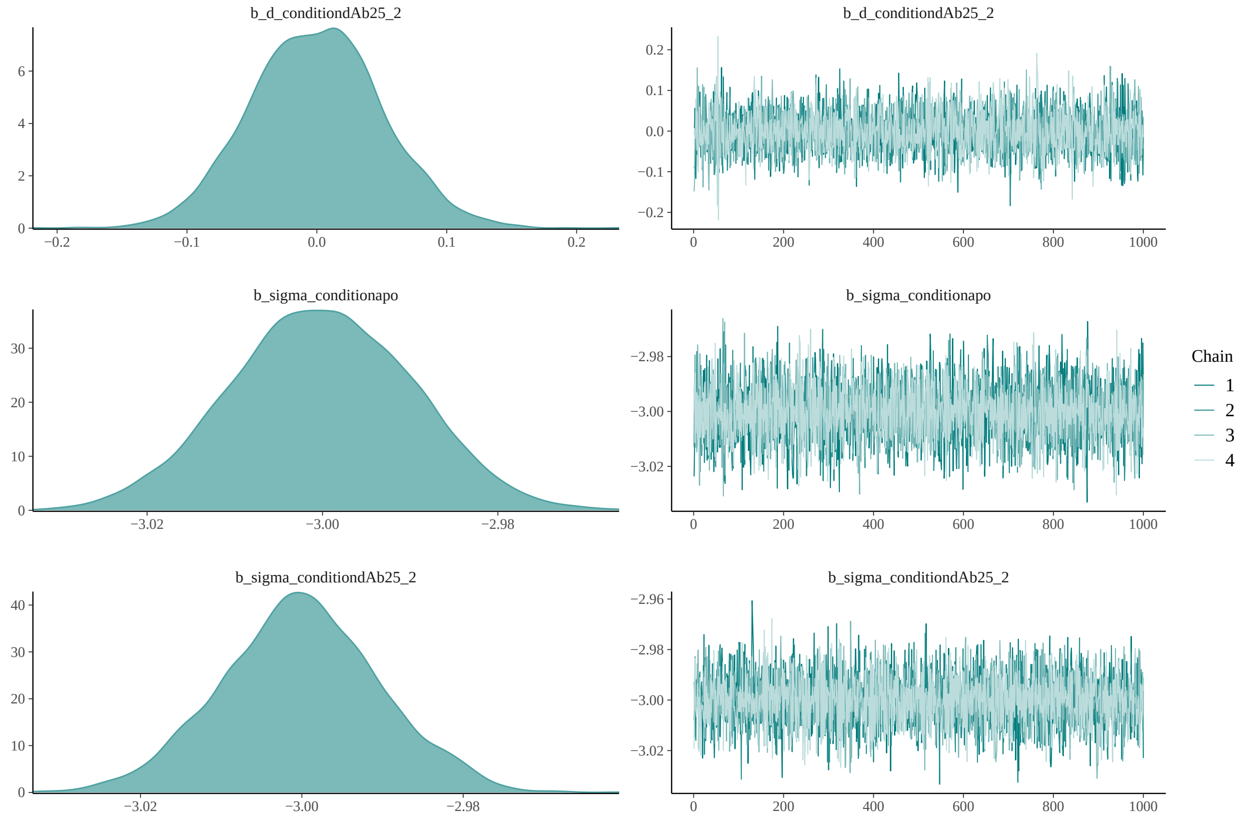

Supplementary Figure S4: **Example trace plots.** Trace plots for example parameters from MCMC runs, convergence is clearly achieved

1.3 Control of error rates in null experiments

To demonstrate that our Bayesian analysis controls false discoveries, we perform a permutation experiment, using the experiment on MBP generated in seven replicates introduced in the main text. The seven MBP samples without any structural variant can be used as a null experiment by partitioning the replicates *falsely* into two conditions. That is three of the samples are labelled condition A and four samples are labelled condition B, arbitrarily. We randomly permute the samples labelled A and B, five times. We then computed the posterior probability that each peptide is perturbed (alternative model). For each permutation this is visualised in a histogram of all the probabilities. We see that the posterior probability is never above 0.05 suggesting excellent control of error rates; that is, we never give confident support to the wrong model. We also check that the probabilities are calibrated by computing the Brier score for each permutation experiment. We plot the Brier scores as a boxplot and see that they are essentially 0, indicating good calibration.

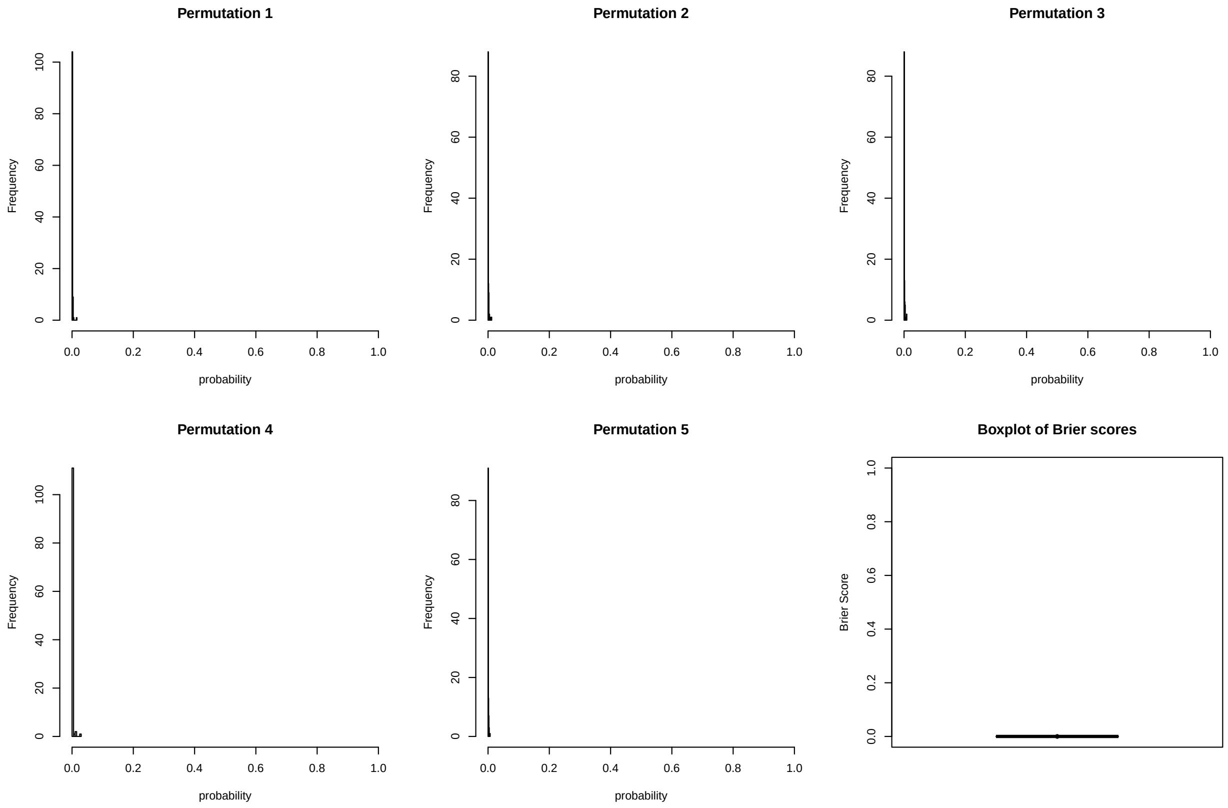

Supplementary Figure S5: **Bayesian analysis controls error rates in null experiments.** (1-5) Histograms for the computed posterior probability of the alternative model. The alternative model is clearly never strongly supported (6) The histogram of Brier scores for each null permutation experiments. The values are close to 0

1.4 Bayesian model selection for HDX

1.4.1 A Weibull model for EX1 kinetics

When a portion of amides undergo correlated deuterium exchange due to simultaneous unfolding and refolding, we observe EX1 kinetics. EX1 kinetics describe different populations of proteins undergoing different hydrogen-deuterium exchange mechanisms. This results in multi-modal deuterated spectra, in which each mode corresponds to a different protein sub-population. This behaviour can be challenging to model. Briefly, at the level of each residue a logistic model is an appropriate model of exchange:

$$y_r = a_r(1 - \exp(-b_r t)), \tag{1}$$

where r indexes residues, b_r denotes the rate constant, a_r the deuterium recovery and t is the time of exposure to heavy water. However bottom-up mass-spectrometry measures peptides and so the observation process is:

$$y_i = \sum_{r \in \mathcal{R}_i} a_r(1 - \exp(-b_r t)). \tag{2}$$

Here i index peptides and $\mathcal{R}_i$ the set of exchangeable residues for peptide i. However, in practice, it is challenging to fit this model because of the number of parameters typically exceeds the number of observations. Therefore, it is required to approximate the kinetics for each peptide. Some suggested models include the following[1,2]:

$$\begin{aligned} \text{Logistic: } y_i &\approx a_i(1 - \exp(b_i t)) \\ \text{Weibull: } y_i &\approx a_i(1 - \exp(b_i t^{q_i})) \\ \text{Sum of logistics: } y_i &\approx a_{i1}(1 - \exp(b_{i1} t)) + a_{i2}(1 - \exp(b_{i2} t)). \end{aligned} \tag{3}$$

A more complex possibility is a non-parametric model $y_i \approx f(t)$, but we have chosen to explore if the simpler models are sufficient to explain the majority of HDX-MS kinetics. Currently, there is no statistical methodology or guidance on determining which approximation to use for statistical testing for peptide-centric HDX-MS data. We are particularly interested in determining the preferred model for data with EX1 kinetics. Here, using Bayesian statistics, we test whether a logistic or Weibull model is a better model of centroided EX1 kinetics (see methods). To perform this analysis, we simulate EX1 kinetics for 100 peptides. The length of each peptide was sampled uniformly between 5 and 15, with the amino acids chosen uniformly at random. The first mode was assumed to have 0 deuterium incorporation, whilst the incorporation for the second mode was sampled uniformly from $[0, 1]$ and the charge state was sampled uniformly between one and eight. Deuterium incorporations measurements were made at $0, 300, 500, 700$ and 1000 seconds post exposure to heavy water. The relative proportion of the second mode was assumed to rise exponentially. For each peptide two replicates were simulated to allow for natural variations. Bimodal spectra were simulated using the natural isotope distributions for that peptide and the centroid computed. To examine whether a logistic or Weibull model was preferred, we used the posterior probability of each model, as well as the leave-one-out expected log predictive density - a measure of out of sample predictive performance of our models (see methods for more details). Figure S6 plots paired boxplots which show that the Weibull model was preferred according to both measures. This is despite the additional penalisation of the Weibull model because of a more diffuse prior.

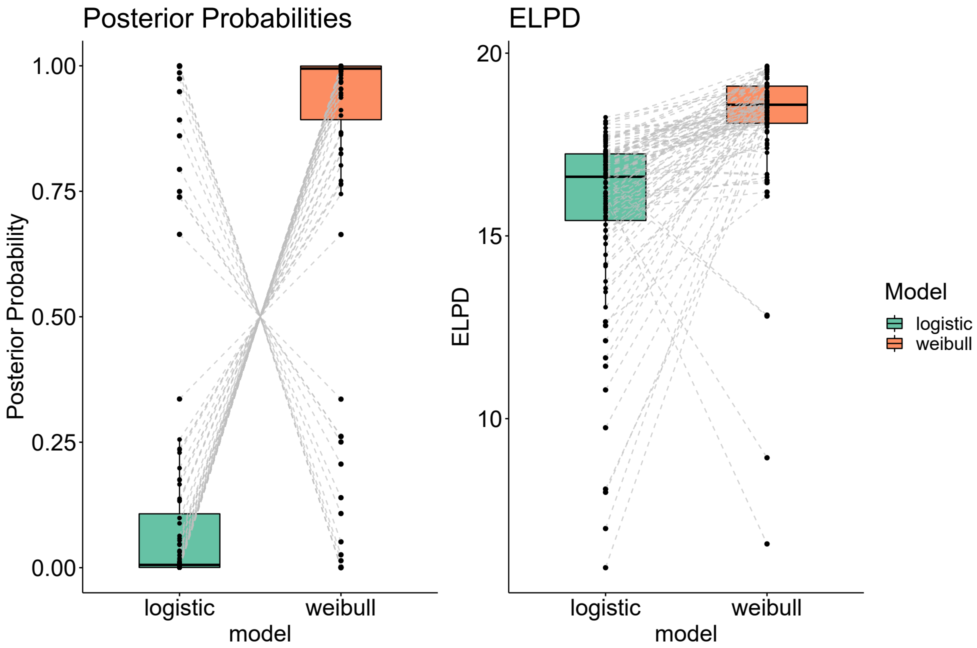

Supplementary Figure S6: **Examining model selection for EX1 kinetics.** Paired boxplots for the two metrics of interest: posterior model probabilities (left) and leave-one-out expected log predictive density (ELPD) (right). Grey lines indicating the simulation pairings, indicating not only preference on average for the Weibull model but the vast majority of peptides.

1.4.2 Model selection for structural spike-in experiment

Having analysed simulated data, we next analyse a structural spike-in experiment, where HDX data on maltose-binding protein (MBP) was generated in seven replicates across four HDX labelling times[3]. Additional experiments were carried out in triplicate for the W169G (tryptophan residue 169 to glycine) structural variant. Here MBP-W169G was spiked into the wild-type MBP sample in $5, 10, 15, 20, 25\%$ proportions, and a further experiment included a 100% mutant sample. All data were analysed on a Agilent 6530 Q-TOF mass spectrometer and raw spectra processed in HDExaminer. Since there are two populations of proteins (either the WT or W169G), these HDX data undergo multi-modal exchange dynamics. This allows us to further test our previous model selection approach in practice. Furthermore, as these experiments are replicated we can also examine a random-effects model which could account for random variations in the plateauing of the HDX kinetics (see methods) across replicates. Thus, there are now three possible models to consider: a logistic model, a Weibull model, a Weibull model with random plateaus. To compare these models formally, we again use posterior model probabilities and the leave-one-out expected log predictive density (ELPD). For brevity, we consider the 10% and 15% spike-in experiments.

Figure S7 shows ternary plots for these three models, where the metrics have been rescaled to indicate relative model preference. Each pointer represents a modelled peptide. In general, the posterior probability suggests a preference for the Weibull model. Occasionally, there is a preference for logistic model with little support for the more complex random plateaus model. The conclusions are somewhat similar when using ELPD, but the random plateau model has a little more

support. This suggests that the preferred model is a Weibull model and the more complex random plateau model is unnecessarily complex but might be useful in predicting new data. Hence, for under-determined HDX-MS data, where there are fewer measured data points than the full kinetic model, Bayesian modelling offers a principled approach to determine an approximate model. In the supplementary material, we also show that our Bayesian approach does not generate false positives in a null permutation experiment.

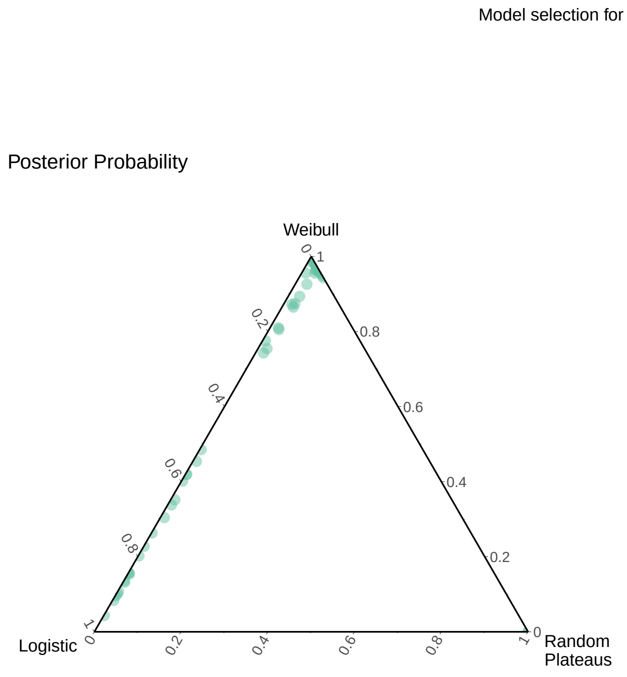

Supplementary Figure S7: **Examining model selection for structural spike-in kinetics.** Ternary plots for the two metrics of interest: posterior model probability (left) and ELPD_{LOO} (right). We consider 3 models: a Logistic model (left), a Weibull model (top) and Random Pleateus (right). The metrics have been rescaled to indicate relative model preference. Each pointer represent the metrics for a modelled peptide. The closer the pointer is to the apex indicating the model, the greater the preference for that model.

1.5 Differential solvent accessibility analysis

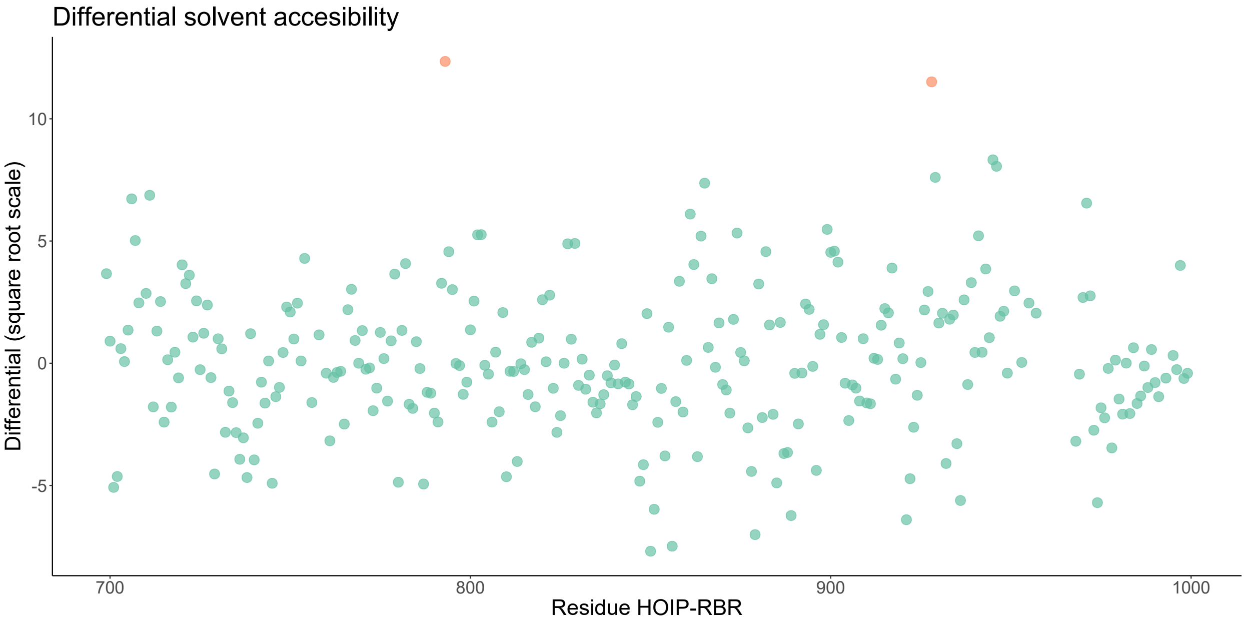

Supplementary Figure S8: **Differential solvent accessibility analysis** Differential solvent accessibility analysis of HOIP-RBR in bound (6SC6) and unbound (5EDV) forms. The difference in solvent accessibility is plotted on the squart-root scale against the HOIP-RBR residue number. Significantly difference are identified by computing the local *false discovery rate* (FDR) and highlighted in orange.

1.6 HOIP-RBR structure and peptides

Here, we plot the structure of HOIP-RBR and highlight peptides referenced in the main manuscript

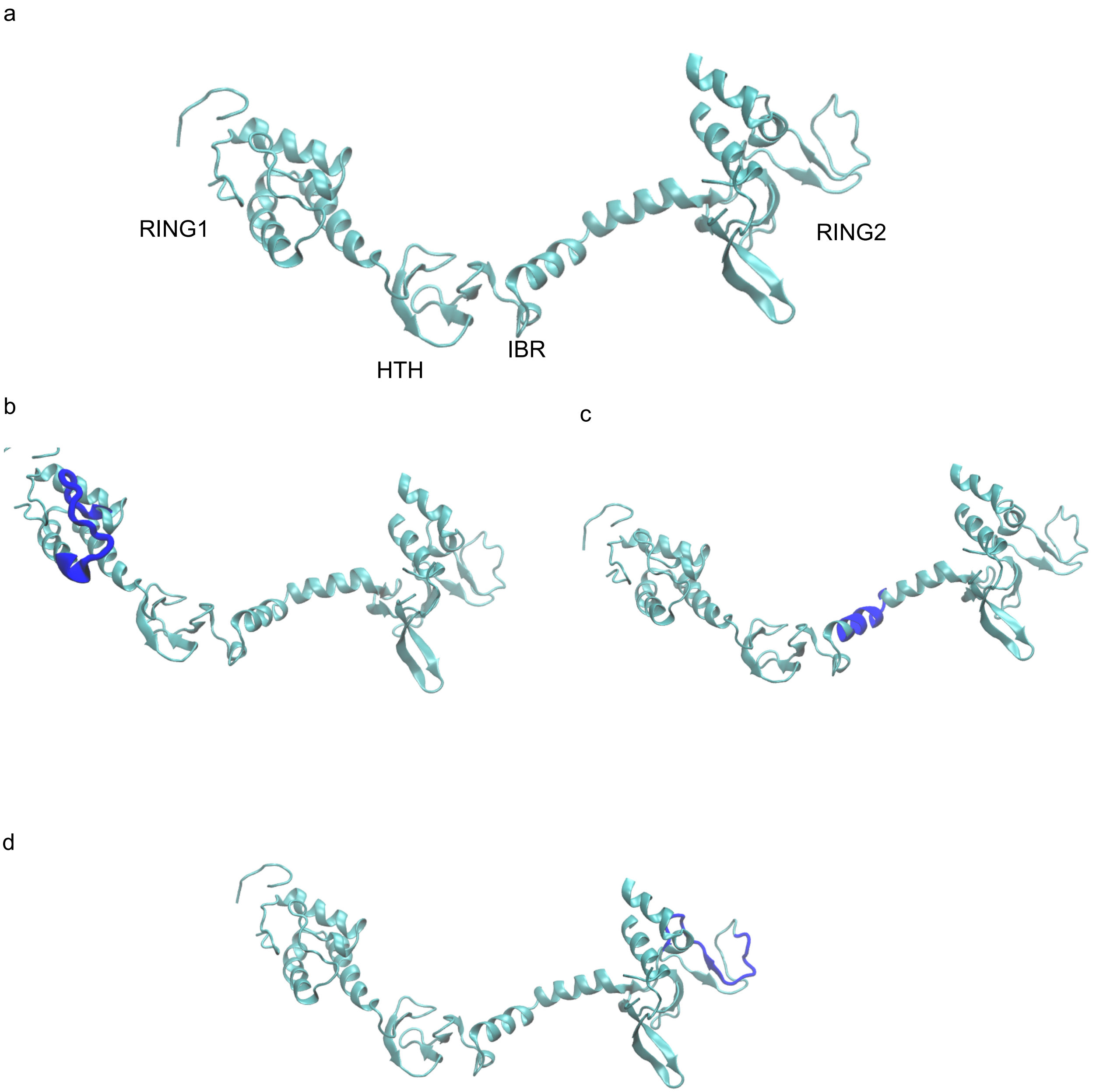

Supplementary Figure S9: **HOIP-RBR structures**(a) HOIP-RBR structure (pdb: 5EDV) (b) peptide 742-758 (c) peptide 844-855 (d) peptide 917-931

1.7 Figure for HOIP-RBR-dAb25

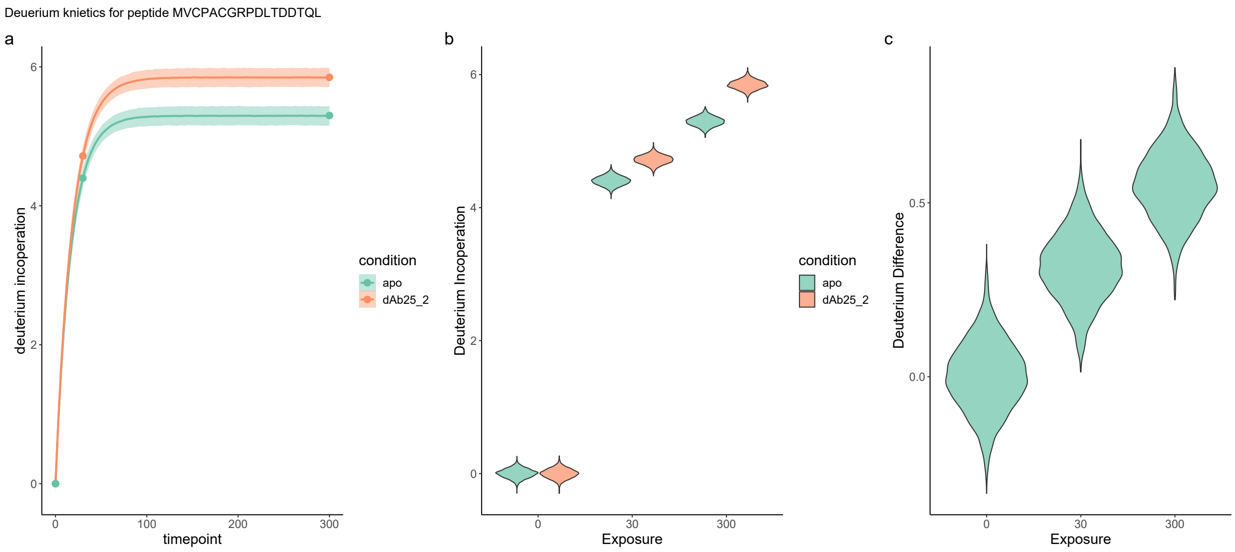

Supplementary Figure S10: **Posterior kinetics for peptide from the Bayesian analysis.** (a) The posterior predictive logistic distribution for peptide across the temporal dimension. (b) Posterior predictive distribution of deuterium incorporation for each state. Violin plots represent uncertainty in the deuterium incorporation. (c) Deuterium differences calculated from the posterior predictive distributions. We can clearly see that the probability that the deuterium difference is greater than 0 is close to 1 at 30 and 300 seconds.

1.8 Figure for HOIP-RBR-dAb3

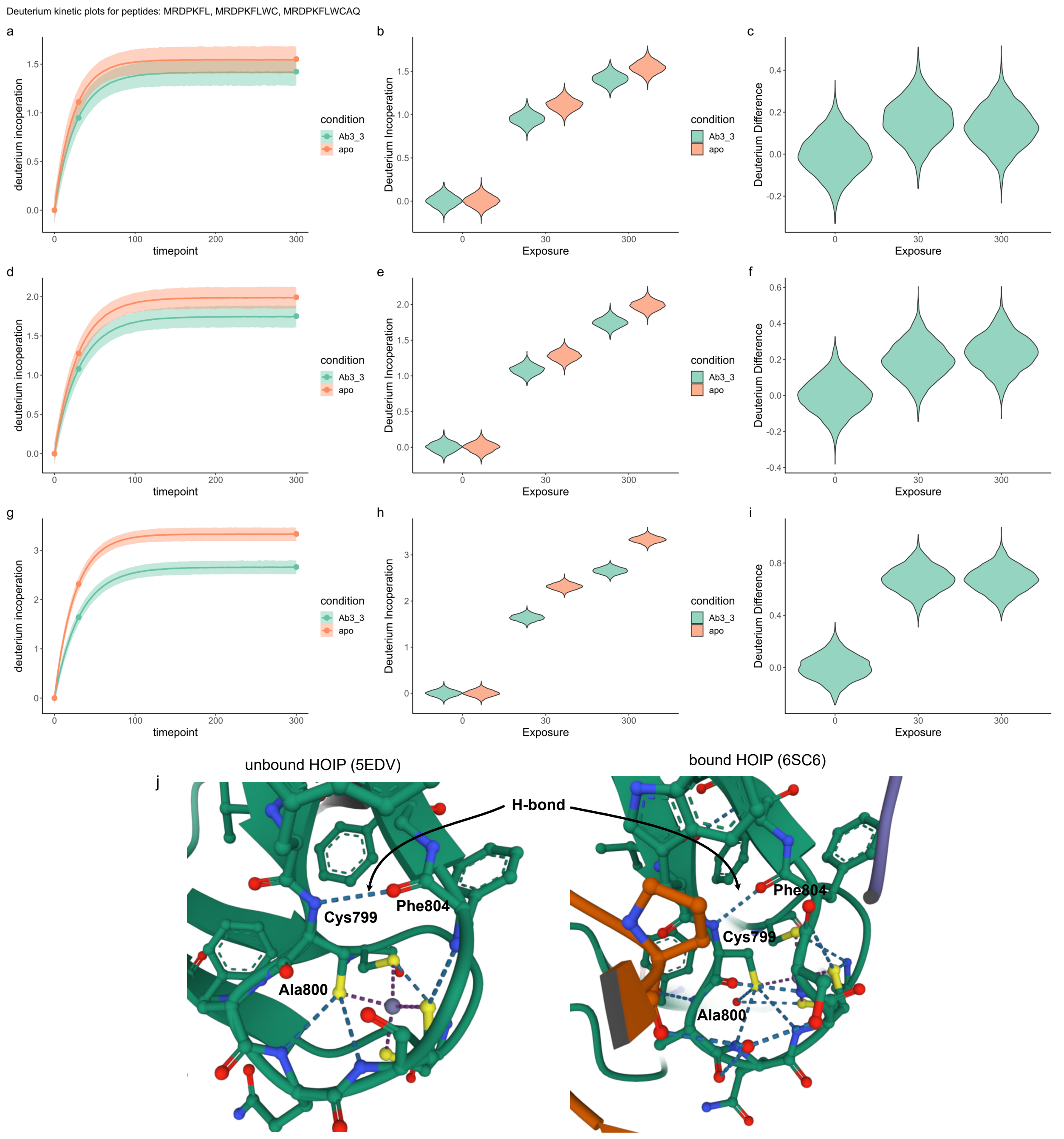

Supplementary Figure S11: **Posterior predictive kinetic plots for three peptides** MRDPKFL is plotted on row 1; MRDPKFLWC on row 2 and MRDPKFLWCAQ on row 3 (a,d,g) The posterior predictive logistic distribution for peptide across the temporal dimension. (b,e,h) Posterior predictive distribution of deuterium incorporation for each state. Violin plots represent uncertainty in the deuterium incorporation. (c,f,i) Deuterium differences calculated from the posterior predictive distributions. Violin plots overlap with zero for exposure times of 30 and 300 seconds. (j) zooms of amides of C799 and A800 on HOIP-RBR in complex with dAb3 dimer (right) and HOIP in complex with the ubiquitin-transfer complex (left). Antigen is coloured in dark green, antibody dimer in brown and purple, elements are coloured using standard colourings. The zinc cation is denoted by a grey sphere and waters are not shown. The corresponding hydrogen bond between C799 and F704 are annotated. Note that A800 does not directly form a hydrogen bond with the dAb3 dimer.

1.9 Agreement between X-ray crystal structure and HDX

dAb interacting residues determined by Tsai et al. [4]	Distance analysis of the co-crystal structure	Solubility analysis	HDX-MS analysis peptide	Probability of deuterium protection
R792	D766 - E809		MRDPKFL, MRDPKFLWC, MRDPKFLWCAQ	0.10850, 0.46475, 1.00000
D793	D766 - E809	D793	MRDPKFL, MRDPKFLWC, MRDPKFLWCAQ	0.10850, 0.46475, 1.00000
Q801	D766 - E809		NA	
C802	D766 - E809		NA	
S803	D766 - E809		NA	
Q822	C817 - W832		EATCPQCHQTF, EATCPQCHQTFC REQLEATCPQCHQTFC	0.01550, 0.01075, 0.02750
R827	C817 - W832		CVRCKRQWEEQHRGRSCE, CVRCKRQWEEQHRGRSCED, CVRCKRQWEEQHRGRSCEDFQN	0.83525, 0.87125, 0.00175
K829	C817 - W832		CVRCKRQWEEQHRGRSCE, CVRCKRQWEEQHRGRSCED, CVRCKRQWEEQHRGRSCEDFQN	0.83525, 0.87125, 0.00175
	R849 - Q858		FQNWKRMNDPEY	0.80750
	R849 - Q858		FQNWKRMNDPEY	0.80750
	R849 - Q858		FQNWKRMNDPEY	0.80750
		R928	RVKKSLHGHHPRCVL	0.04625

Supplementary Table S1: A summary of the correspondence between the Bayesian analysis of HDX-MS data and the co-crystal structure. Distance analysis is performed at eight Å. Solubility analysis is performed as described in the supplement. HDX-MS analysis peptide indicates the measured peptide. NA is used to indicate no peptide with exchangeable residues was measured for that residue.

1.10 HDX-MS of BRD4

A 6xHis-BRD4(1-477) construct was expressed and purified as previously described [5]. For HDX labelling experiments, 2.5 μM stock of BRD4 protein (12.5 pmol) was prepared by dilution in a reference buffer of 50 mM MOPS, 150 mM NaCl, pH 7.2. The inhibitor-bound and apo forms were prepared by the addition of i-BET151 (for a final concentration of 25 μM) or an equivalent volume of DMSO (2% final concentration) and pre-incubated for at least 30 min at 1°C.

The reaction was initiated by a 12-fold dilution of 5 μL protein sample (12.5 pmol) in labelling buffer (50 mM MOPS, 150 mM NaCl, pD 7.2 (pHread = 6.8 with standard calomel electrode)) at 20°C using an automated sample handling workflow (LEAP HDX PAL, Trajan Scientific). Labelling times were sampled at 0, 15, 60, 600, 3600, 14400 s in triplicate. Protein samples were quenched and denatured by an equal volume of quench solution (6 M guanidine hydrochloride, 400 mM sodium phosphate pH 2.2, 2% formic acid) for 1 min at 1°C and immediately injected onto an immobilized nepenthesin-2 column (2.1 mm x 20 mm, Affipro, CZ). The resultant peptides collected on a pre-column trap (UPLC BEH C18 Vanguard, Waters) for 4 min with 0.2% formic acid, 0.03% TFA in H2O at a flow rate of 100 μL/min. Peptides were then eluted by liquid chromatography (1.7 μm UPLC BEH C18 column, 1.0 x 50 mm dimensions, 130Åpore size, Waters) for 12 min at a flow rate of 20 μL/min at 0°C, over a gradient of 11-40% of 0.2% formic acid in MeCN before ramping to 98% for a further 3 min, and a 4 min sawtooth gradient cleaning cycle. A LeuEnk and GluFib lock solution was co-injected as an internal standard. Data was acquired on a Synapt G2-Si high definition mass spectrometer (Waters) in the data-independent HDMS acquisition mode. For fully labelled control experiments, BRD4 was labelled for 1 h in 6 M d5-guanidine deuteriochloride diluted in labelling buffer, before quenching as described above. Samples for 100% D control experiments were handled manually.

Peptide mapping experiments were completed in a separate experiment with a 25 pmol injection of protein diluted in the unlabelled reference buffer, and subsequently treated as above, using a HDMSe acquisition mode. Peptide lists from the peptide mapping experiments were generated in Protein Lynx Global Server 3.0 software. The peptide lists generated were subsequently imported into HDExaminer v2.5.0 (Sierra Analytics, Modesto, CA) and subject to further filtering (peptide length < 25; PLGS Score > 6.5; Products per amino acid > 0.3; Δ ppm $< \pm$ 10) and analysed to determine deuterium uptake. Only peptides containing data with adequate intensity and reliable isotope distributions for all timepoints and both states were preserved.

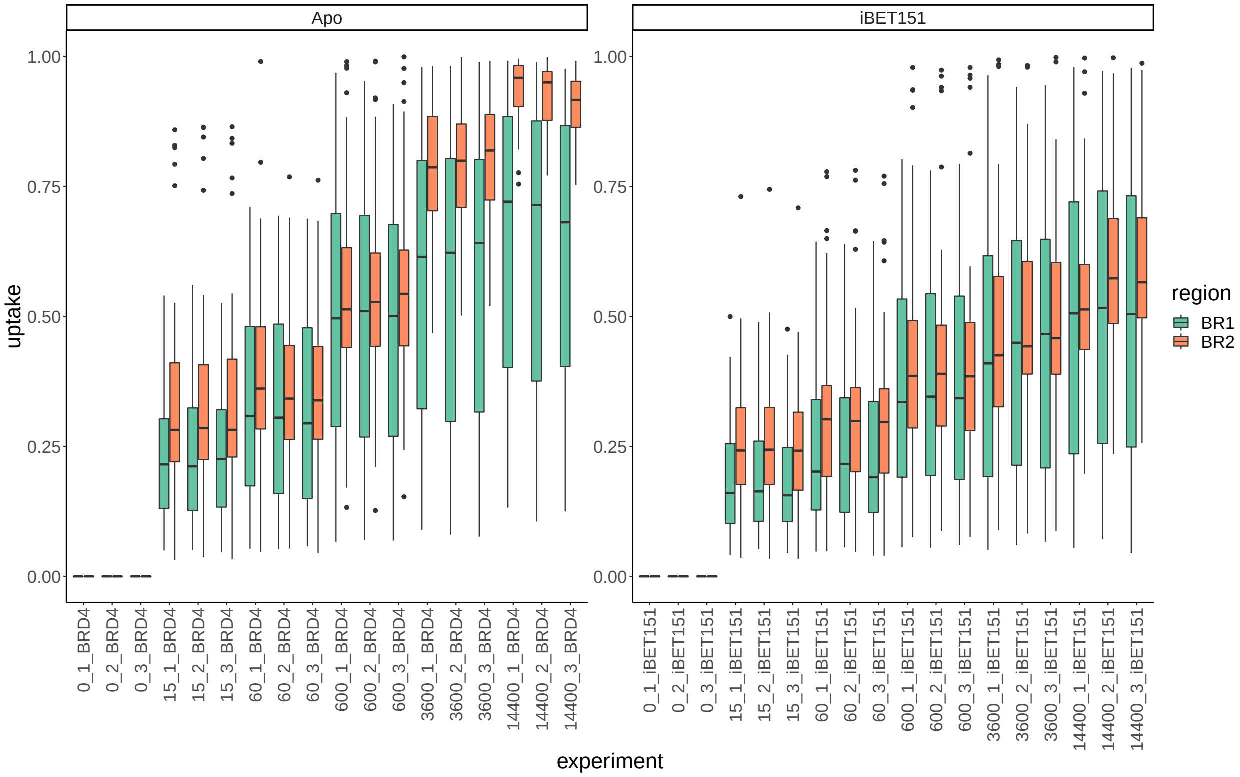

Supplementary Figure S12: **BRD4 relative deuterium uptake in Bromodomains** (a) Boxplots showing the distribution of deuterium uptake across the two bromodomains (BR1 and BR2).

References

[1] Palaniappan Sevugan Chetty et al. **2009:** “Helical structure and stability in human apolipoprotein AI by hydrogen exchange and mass spectrometry”. In: *Proceedings of the National Academy of Sciences* 106.45, pp. 19005–19010.

[2] Oliver M Crook, Chun-wa Chung, and Charlotte M Deane **2022:** “Empirical Bayes functional models for hydrogen deuterium exchange mass spectrometry”. In: *Communications biology* 5.1, pp. 1–10.

[3] Tyler S Hageman and David D Weis **2019:** “Reliable identification of significant differences in differential hydrogen exchange-mass spectrometry measurements using a hybrid significance testing approach”. In: *Analytical chemistry* 91.13, pp. 8008–8016.

[4] Yi-Chun Isabella Tsai et al. **2020:** “Single-domain antibodies as crystallization chaperones to enable structure-based inhibitor development for RBR E3 ubiquitin ligases”. In: *Cell chemical biology* 27.1, pp. 83–93.

[5] Romain Gosmini et al. **2014:** “The discovery of I-BET726 (GSK1324726A), a potent tetrahydroquinoline ApoA1 up-regulator and selective BET bromodomain inhibitor”. In: *Journal of medicinal chemistry* 57.19, pp. 8111–8131.
